# Synthesis, Fluorine-18 Radiolabeling, and In Vivo PET Imaging of a Hydrophilic Fluorosulfotetrazine

**DOI:** 10.3390/ph16050636

**Published:** 2023-04-22

**Authors:** Jason Beaufrez, Stéphane Guillouet, Yann Seimbille, Cécile Perrio

**Affiliations:** 1 UAR 3408, CNRS, CEA, Unicaen, Cyceron, Bd Henri Becquerel, 14074 Caen, France; 2 Department of Radiology & Nuclear Medicine, Erasmus MC, University Medical Center Rotterdam, Wytemaweg 80, 3015 CN Rotterdam, The Netherlands

**Keywords:** tetrazine, fluorine-18, PET imaging, hydrophilicity, bioconjugation, pre-targeting, sultone, sulfonic acid salt

## Abstract

The development of ^18^F-fluorotetrazines, suitable for the radiolabeling of biologics such as proteins and antibodies by IEDDA ligation, represents a major challenge, especially for pre-targeting applications. The hydrophilicity of the tetrazine has clearly become a crucial parameter for the performance of in vivo chemistry. In this study, we present the design, the synthesis, the radiosynthesis, the physicochemical characterization, the in vitro and in vivo stability, as well as the pharmacokinetics and the biodistribution determined by PET imaging in healthy animals of an original hydrophilic ^18^F-fluorosulfotetrazine. This tetrazine was prepared and radiolabelled with fluorine-18 according to a three-step procedure, starting from propargylic butanesultone as the precursor. The propargylic sultone was converted into the corresponding propargylic fluorosulfonate by a ring-opening reaction with ^18/19^F-fluoride. Propargylic ^18/19^F-fluorosulfonate was then subject to a CuACC reaction with an azidotetrazine, followed by oxidation. The overall automated radiosynthesis afforded the ^18^F-fluorosulfotetrazine in 29–35% DCY, within 90–95 min. The experimental LogP and LogD_7.4_ values of −1.27 ± 0.02 and −1.70 ± 0.02, respectively, confirmed the hydrophilicity of the ^18^F-fluorosulfotetrazine. In vitro and in vivo studies displayed a total stability of the ^18^F-fluorosulfotetrazine without any traces of metabolization, the absence of non-specific retention in all organs, and the appropriate pharmacokinetics for pre-targeting applications.

## 1. Introduction

**Figure 1:**
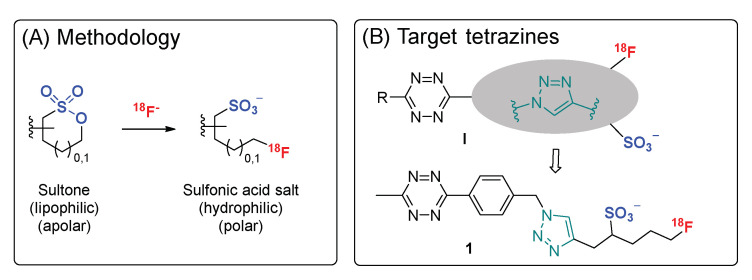
Objective of the study, with (**A**) the radiofluorination strategy by sultone ring opening, and (**B**) the general chemical structure of the hydrophilic target [^18^F]fluorosulfotetrazines **I**, and the lead compound **1**.

1,2,4,5-Tetrazines have found applications in many fields, especially in the bioconjugation and radiolabeling of bio-active vectors for imaging and therapy [1,2,3,4,5,6,7]. They are essential reagents for bioorthogonal approaches via the inverse electron demand Diels–Alder (IEDDA) reaction with a dienophile, such as trans-cyclooctene (TCO). The IEDDA reaction, which leads to inert dinitrogen as the sole by-product, has the advantage of having fast kinetics (102–106 M-1s-1) and biocompatibility, making this reaction suitable for the functionalization of biologics. It has become the method of choice for the development of imaging and theranostic agents that are based on proteins and antibodies. The IEDDA reaction also represents one of the most popular reactions for pre-targeting strategies, allowing for the use of short-half-life radioisotopes such as fluorine-18 (t_1/2_ = 109.8 min) for the in vivo radiolabelling of antibodies with long pharmacokinetics [6]. Fluorine-18 is very attractive due to its ideal physical properties for imaging (E_γ_^+^ = 0.63 MeV, γ^+^ range in a tissue < 2.4 mm). Thus, numerous ^18^F-tetrazines, obtained by aliphatic or (hetero)aromatic radiofluorination, or by fluorine-heteroatome (Al, B, S, Si) bond formation, have been reported in recent years [8,9,10,11,12,13,14,15,16,17,18,19,20,21,22]. However, the success of pre-targeting strategies is still limited. One of the main reasons for this is the in vivo behavior of the ^18^F-terazines, including non-specific retention and radiometabolization. Lastly, Herth and co-workers clearly demonstrated that hydrophilicity was a crucial parameter for the performance of the ^18^F-tetrazines in in vivo chemistry [11]. For two tetrazines that displayed the same rate constant in the reaction with TCO, the best results in pre-targeting were obtained with the most hydrophilic tetrazine. Currently, the hydrophilicity of ^18^F-tetrazines, evaluated using in silico calculation of the partition coefficient at physiological pH (clogD_7.4_), was brought by PEG chains [11,17], sugars moieties [9,10], or iminodiacetic acid groups [13,14,16]. Another well-established strategy to enhance the hydrophilic character of organic molecules is to introduce a sulfonic acid function [23]. We previously reported that ^18^F-fluorosulfonic acid salts were easily obtained by the direct radiofluorination of sultone precursors (Figure 1A) [24]. This ring-opening reaction approach has the advantage of smooth reaction conditions and ease of separation of the polar hydrophilic ^18^F-sulfonic product from the apolar hydrophobic sultone precursor. Consequently, based on this sultone radiofluorination methodology, we designed the [^18^F]fluorosulfotetrazines **I** as a new class of IEDDA partners (Figure 1B). This class of tetrazines also contains a triazole ring that is known to be stable in vivo, and facilitates aqueous solubility [25]. In this study, we report the synthesis and radiosynthesis of the lead fluorosulfotetrazine **1** for proof-of-concept, as well as its characterization including hydrophilicity, in vitro and in vivo stability, and pharmacokinetics via PET imaging.

## 2. Results and Discussion

### 2.1. Synthesis of the Fluorosulfotetrazine ***1***

**Scheme 1:**
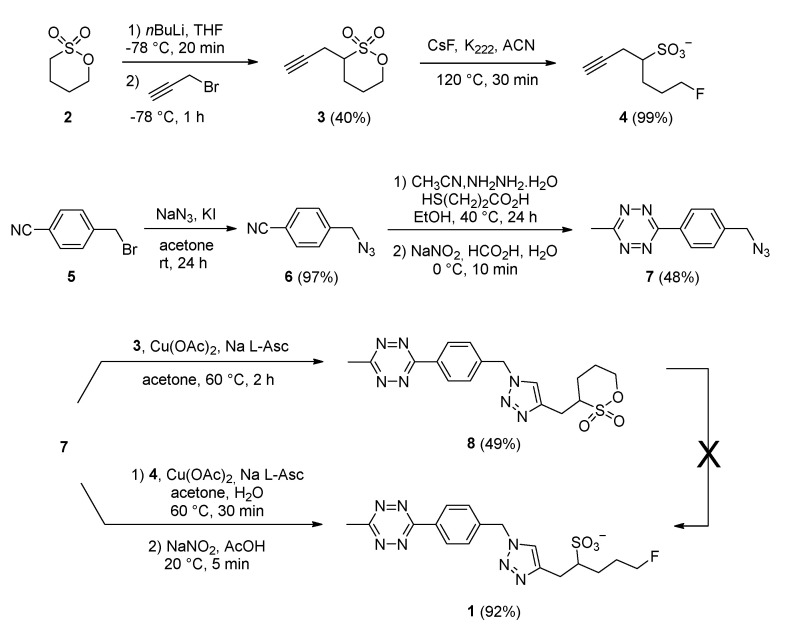
Synthesis of tetrazinesultone **8** and fluorosulfotetrazine **1**.

The synthesis of the fluorosulfotetrazine **1** was designed in a convergent manner to link the sultone ring (labeling precursor) or the fluorosulfochain (final pattern) to the tetrazine moiety, while creating the triazole ring via a CuAAC reaction [11]. According to this strategy, the retrosynthetic approach rapidly led to propargylic butanesultone **3** and azidotetrazine **7** as key intermediates (Scheme 1). Propargylic butanesultone **3** was obtained with a 40% yield by the alkylation of commercially available butanesultone **2** with propargyl bromide, after deprotonation with nBuLi in THF at −78 °C. Fluorination of propargyl butanesultone **3** was carried out with cesium fluoride in acetonitrile at 120 °C for 30 min in the presence of Kryptofix-222^®^ (K_222_). The resulting fluorosulfonate **4** was obtained in a quantitative yield. In parallel, 4-(bromomethyl)benzonitrile **5** was treated with sodium azide and potassium iodide in acetone at room temperature for 24 h to afford 4-(azidomethyl)benzonitrile **6**, at a 97% yield. The azidobenzonitrile **6** underwent a modified Pinner reaction with acetonitrile and hydrazine hydrate in the presence of 3-mercaptopropionic acid in ethanol at 40 °C for 24 h [26]. After oxidation with sodium nitrite and formic acid in a water/ethanol mixture, the azidotetrazine **7** was isolated with a 48% yield. Azidotetrazine **7** and propargylic butanesultone **3** were subject to a CuAAC reaction in the presence of Cu(OAc)_2_ and sodium ascorbate (Na L-Asc) in acetone at 60 °C for 2 h, resulting in tetrazinesultone **8** at a 49% yield. All of the attempts to convert **8** to fluorosulfotetrazine **1** by direct fluorination failed. Fluorosulfotetrazine **1** was finally obtained with a 92% yield according to a two-step sequence by the CuAAC reaction between azidotetrazine **7** and propargylic fluorosulfonate **4** at 60 °C for 30 min, followed by oxidation with sodium nitrite and acetic acid at 20 °C for 5 min.

### 2.2. IEDDA Reaction of the Fluorosulfotetrazine ***1*** with TCO Reagent ***9***

**Scheme 2:**
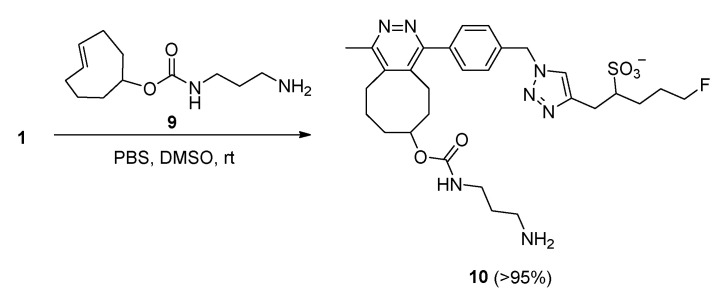
IEDDA reaction of the fluorosulfotetrazine **1** with TCO reagent **9**.

The reactivity of the fluorosulfotetrazine **1** was checked in a model bioorthogonal IEDDA reaction with TCO reagent **9** (Scheme 2). The reaction was carried out in PBS containing 5% DMSO, and was found to be instantaneous at room temperature, as demonstrated by the discoloration of the reaction mixture when both reagents were added. LC–MS analysis confirmed that the tetrazine **1** was immediately consumed in presence of TCO reagent **9**, resulting in a reaction conversion of over 95%, and the identification of pyridazine adduct **10** (see Supplementary Materials for LC–MS spectrum). Although the rate constants for the IEDDA reactions with methyltetrazines such as **1** were not the highest compared to those for reactions with the pyridino or hydrogeno analogues [11], the reactivity of tetrazine **1** toward TCO remained highly notable.

### 2.3. Development of the Radiosynthesis of [^18^F]Fluorosulfotetrazine [^18^F]***1***

**Scheme 3:**
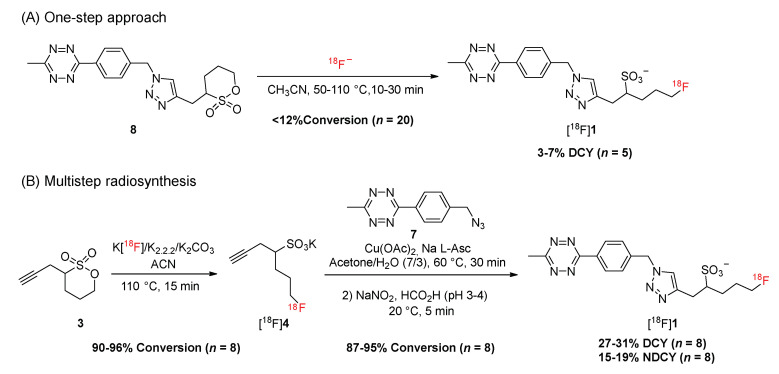
Radiosyntheses of [^18^F]fluorosulfotetrazine [^18^F]**1** according to one-step (**A**) or multistep (**B**) approach.

The radiosynthesis of [^18^F]**1** was first performed manually, using a low amount of starting radioactivity (<185 MBq, 5 mCi). The most attractive strategy to obtain [^18^F]**1** was the one-step approach via radiofluorination of the tetrazinesultone **8** (Scheme 3A). We initially attempted to convert the tetrazinesultone **8** into [^18^F]fluorosulfotetrazine [^18^F]**1** under standard conditions using the K^18^F/K_222_/K_2_CO_3_ complex in ACN, DMF, or DMSO. However, despite our efforts to adapt the amount of K_2_CO_3_ (0.5–10 mg), K_222_ (10–25 mg), temperature (50–130 °C), and reaction time (10–30 min), the radiochemical yields remained below 5%. The replacement of K^18^F by Cs^18^F or TBA^18^F did not improve the conversions. The optimum radiochemical yields reached only 7%, using the “non-basic” [^18^F]fluoride purification method with potassium triflate (10 mg) and K_2_CO_3_ (0.5 mg) [12]. These results were in accordance with the well-admitted difficulty of aliphatic radiofluorination from tetrazine substates [13]. Therefore, we decided to adopt a multistep strategy, starting with the radiofluorination of propargylic sultone **3** (Scheme 3B). The radiofluorination of propargylsultone **3** was carried out with K^18^F/K_222_/K_2_CO_3_ in acetonitrile at 110 °C for 15 min, in 90–96% conversion (*n* = 8, see Supplementary Materials for TLC and HPLC characterizations). The resulting propargylic [^18^F]fluorosulfonate [^18^F]**4** was isolated by SPE using a Sep-Pak^®^ Light QMA, and recovered in acetone at >95%. After elimination of the solvent by distillation under a nitrogen flow, the [^18^F]**4** was treated with azidotetrazine **7**, Cu(OAc)_2_ and Na L-Asc in a 7:3 acetone/water mixture at 60 °C for 30 min, then with NaNO_2_ and formic acid at 20 °C for 5 min. [^18^F]Fluorosulfotetrazine [^18^F]**1** was obtained from the propargylic [^18^F]fluorosulfonate [^18^F]**4** at a 87–95% conversion rate (*n* = 8).

### 2.4. Automated Radiosynthesis of [^18^F]Fluorosulfotetrazine [^18^F]***1***

**Figure 2:**
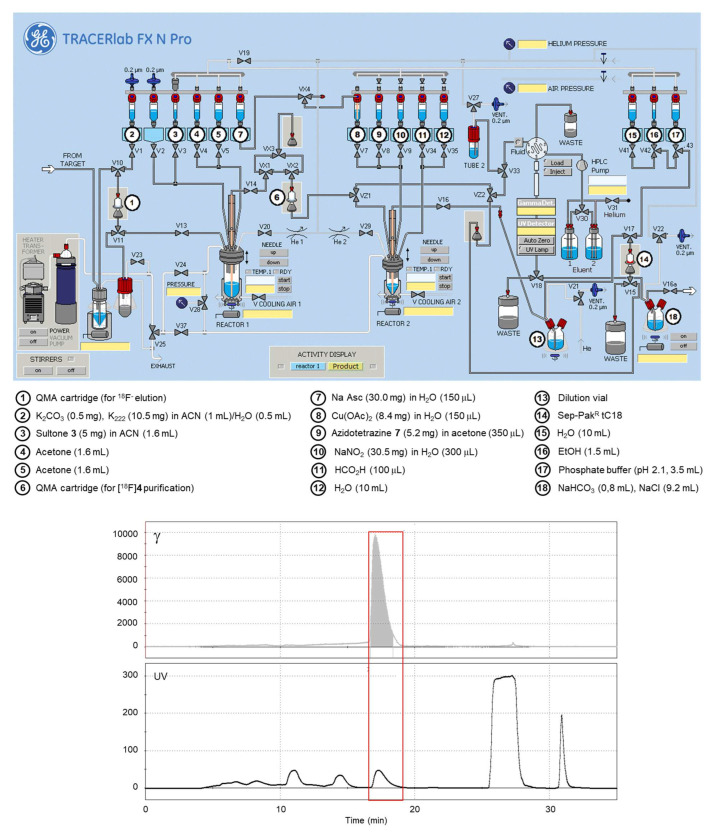
The overall automated process for the radiosynthesis of [^18^F]**1**, and semi-preparative HPLC chromatograms with γ (**up**) and UV (**down**) detection.

The overall multistep radiosynthesis of [^18^F]**1** was transposed on the GE TRACERlab FX NPro module. The reaction conditions (masses of precursors and reagents, solvents, reaction times, and temperatures) used in the manual process were globally retained, but adaptations for intermediate purification were required (Figure 2). [^18^F]Fluoride was recovered from the cyclotron, trapped on an anion exchange resin QMA cartridge (①) and eluted to reactor 1 using a solution of K_2_CO_3_ and K_222_ in acetonitrile and water (②). The K [^18^F]F/K_222_/K_2_CO_3_ complex formed was dried by azeotropic distillation before addition of the sultone precursor **3** in acetonitrile (③). Radiofluorination was carried out at 105 °C for 15 min, and then acetonitrile was removed under reduced pressure and replaced by acetone (④ and ⑥). The resulting propargylic [^18^F]fluorosulfonate [^18^F]**4** in acetone was passed onto a Sep-Pak^®^ Light QMA (⑤). The eluate containing [^18^F]**4** was recovered in reactor 2 and concentrated under reduced pressure, in order to recover about 350 μL of solution. In parallel, Na L-Asc solution (⑦) was added to the aqueous copper acetate solution (⑧). The resulting mixture was stirred with a helium flow for 20 s, then introduced into the reactor 2. The azidotetrazine **7** in acetone (⑨) was then transferred into reactor 2, and the CuAAC reaction was performed at 60 °C for 25 min. The CuAAC reaction was followed by the oxidation step at 25–30 °C for 5 min, after subsequent addition of sodium nitrite in water (⑩) and formic acid (⑪). The resulting final mixture was transferred into the large volume vial (⑬) for dilution through the addition of water (⑫). The dilute crude mixture was passed through a pre-conditioned Sep-Pak^®^ tC18 Plus Long Environmental (⑭). After washing with water (⑮), the product was eluted with EtOH (⑯), and the ethanolic fraction was diluted in phosphate buffer (⑰) before injection into a semi-preparative reverse phase HPLC for purification. Both UV and γ detection were monitored, and a 72/28 mixture of phosphate buffer (pH 2.1)/ethanol was used as the eluent. [^18^F]**1** was collected into the HPLC flask containing physiological serum and sodium bicarbonate (⑱), in order to obtain a pH range of 6.8–7.1 and a maximum 9–9.5% ethanol concentration. Finally, [^18^F]**1** was directly obtained in the formulation solution ready for injection. The overall process led to [^18^F]fluorosulfotetrazine [^18^F]**1** at a 29–35% (*n* = 8) decay-corrected yield within 90–95 min.

### 2.5. Quality Control

**Figure 3:**
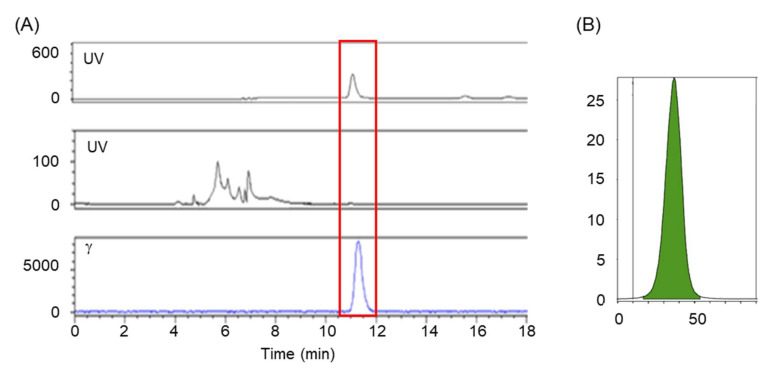
Quality control of [^18^F]**1**, (**A**) using analytical HPLC (top, UV chromatogram of tetrazine **1** for reference; middle, UV chromatogram of [^18^F]**1**; bottom, γ chromatogram of [^18^F]**1**), and (**B**) radioTLC of [^18^F]**1**.

A quality control was carried out using analytical HPLC, with both UV and γ detection, to confirm the identity of the radiotracer, to determine chemical and radiochemical purities, and to calculate the molar activity (Figure 3A). The identity of the radiotracer [^18^F]**1** was unambiguously confirmed, due to the same retention times of the radioactive peak of [^18^F]**1** and the UV peak of the non-radioactive tetrazine **1**. [^18^F]**1** was obtained with chemical and radiochemical purities of >91% and >99%, respectively, and a molar activity of 165 ± 25 MBq/µmol (4.4 ± 0.6 mCi/µmol). The radioTLC analysis of [^18^F]**1** also revealed a single peak, confirming the high radiochemical purity of [^18^F]**1** (Figure 3B).

### 2.6. LogP and LogD_7.4_ Measurements

The hydrophilicity of [^18^F]1 was evaluated through LogP and LogD_7.4_ measurements, using a standard shake flask protocol. The LogP and LogD_7.4_ values for [^18^F]**1** were −1.27 ± 0.02 and −1.70 ± 0.02 (*n* = 6), respectively, demonstrating a high hydrophilicity as expected.

### 2.7. In Vitro Stability Studies

**Figure 4:**
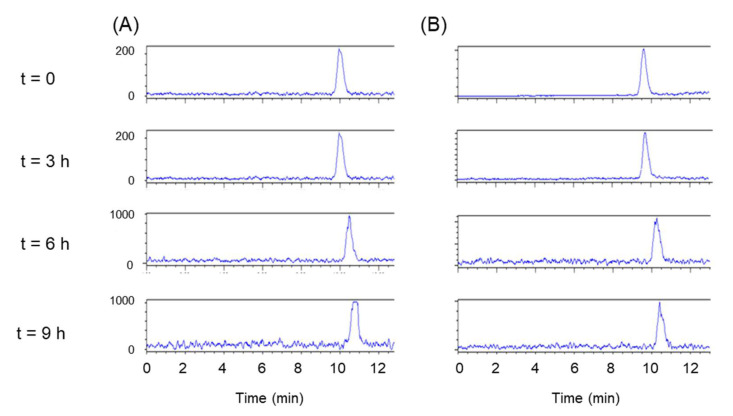
RadioHPLC chromatograms of the stability studies of [^18^F]**1**, (**A**) in the formulated solution, and (**B**) in PBS buffer (pH 7.4).

The stability of [^18^F]**1** was determined via analytical radioHPLC for up to 9 h. [^18^F]**1** was found to be totally stable in the formulated solution, as well as in PBS buffer (pH 7.4), with only intact [^18^F]**1** detected at all time points (Figure 4).

### 2.8. In Vivo Biodistribution Using PET/MR in Mice

**Figure 5:**
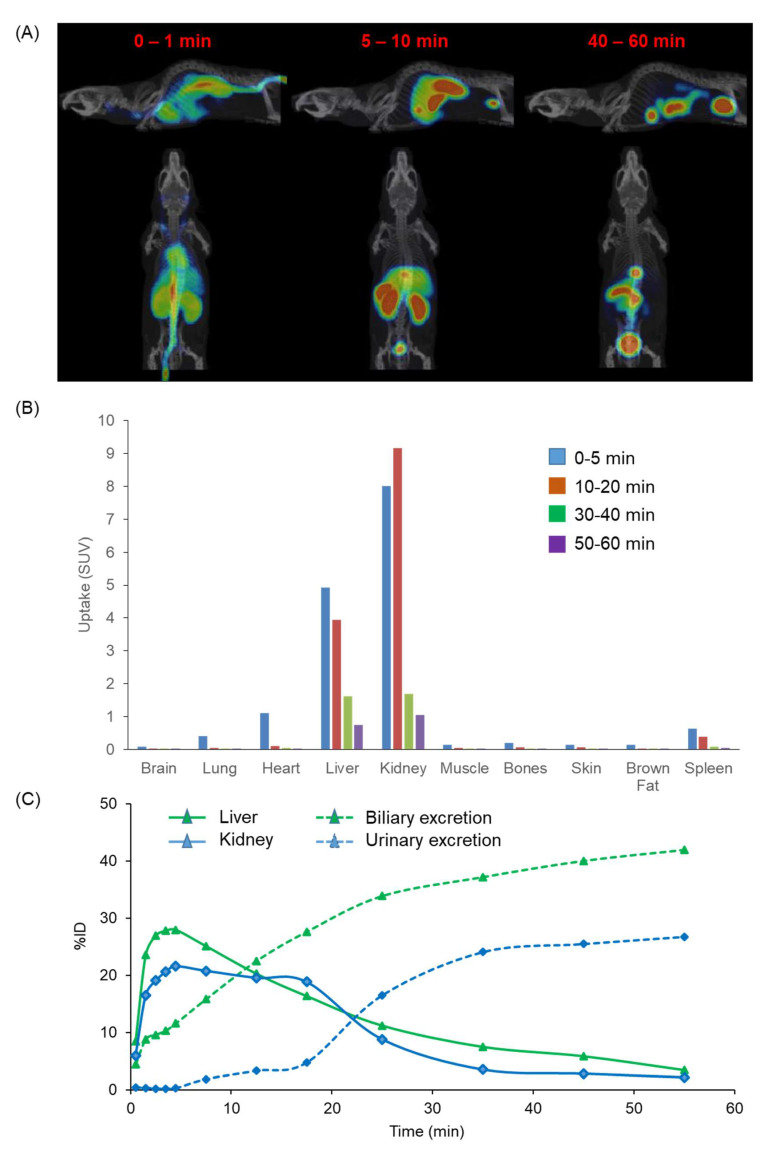
The biodistribution of [^18^F]**1** in healthy mice via PET imaging. (**A**) Representative whole-body PET images; (**B**) tissue distribution; (**C**) time-activity curves (TAC) for elimination pathways.

The formulated [^18^F]1 was administered intravenously to male SWISS mice, and dynamic PET acquisition was performed over 60 min. Figure 5 shows fast perfusion in blood circulation over 2 min, and fast elimination of [^18^F]**1** with only 32% of radioactivity (decay corrected) remaining at 60 min. Five minutes post-injection, a high kidney uptake was observed, which reached a plateau after 10–20 min, and drastically decreased from 30 to 60 min. To a lesser extent, an elevated liver uptake was also visible at 5 min, which decreased steadily throughout the 60 min dynamic image acquisition. The time-activity curves indicated that [^18^F]**1** was eliminated quickly, mainly through both urinary and biliary routes (29% and 39%, respectively, at 60 min). The elimination route of [^18^F]**1** via the renal pathway confirmed its hydrophilic character. However, accumulation in the liver suggests that structural modifications to increase its hydrophilicity may be necessary, in order to obtain better contrast in the abdominal region. Figure 5 does not display any non-specific retention of the radiotracer in the main organs and tissues (i.e., lung, heart, spleen, muscle, skin, and fat), with SUV values below 0.2 from 10 to 60 min. No brain penetration of [^18^F]**1** was detected in accordance with the hydrophilic properties of [^18^F]**1**. No accumulation of radioactivity was observed in bones, precluding the radiodefluorination of [^18^F]**1**.

### 2.9. Radiometabolite Analysis of Mouse Plasma Samples

**Figure 6:**
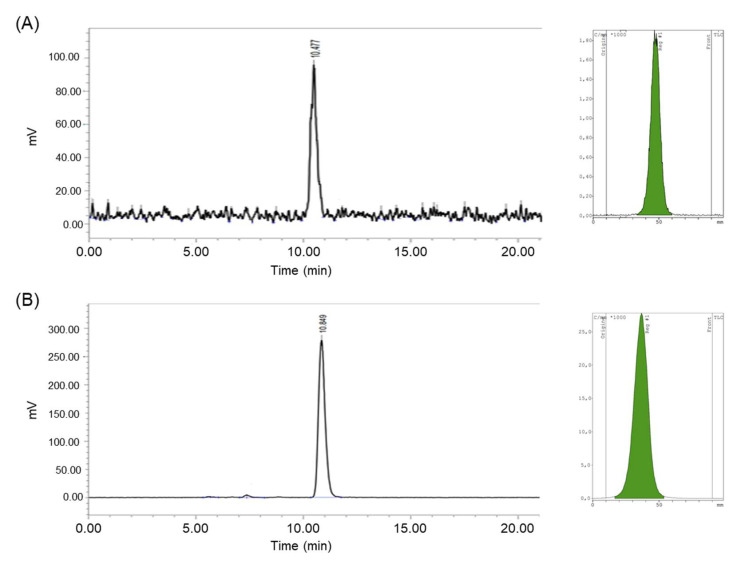
RadioHPLC (left) and radioTLC (right) profiles of (**A**) mouse plasma sample 30 min post-injection of [^18^F]**1**, and (**B**) formulated [^18^F]1 at the end of radiosynthesis.

The in vivo stability of [^18^F]**1** was further examined using radioTLC and radioHPLC analyses of mouse plasma samples collected at 30 min post-injection. As shown in Figure 6, the parent radioactive tetrazine [^18^F]**1** was the only radioactive compound detected with a retention time on the HPLC (t*_R_*~10.4 min) and a retention factor on the TLC (R_f_ = 0.36–0.43) that were similar to those obtained for the quality control. No obvious radiometabolite peak emerged, suggesting that [^18^F]**1** has excellent in vivo stability within 30 min post-injection. This finding corroborates the absence of in vivo radiodefluorination checked with PET imaging, and it points out that no radiometabolite interfered with [^18^F]**1** in the pre-targeting experiments.

## 3. Materials and Methods

### 3.1. Chemical Syntheses

#### 3.1.1. General

All of the commercial reagents were used without further purification. The solvents were dried with appropriate desiccants and distilled prior to use, or were obtained in anhydrous form from commercial suppliers. Silica gel (60, 230–400 mesh or 70–230 mesh from Merck) was used for column chromatography. Celite^®^ 545 was purchased from Sigma-Aldrich. The reactions were monitored using thin layer chromatography on silica gel pre-coated aluminum plates. UV light at 254 nm or KMnO_4_ stains were used to visualize the TLC plates. ^1^H, ^13^C, and ^19^F NMR spectra were recorded using a Bruker Avance spectrometer instrument operating at 500, 126, and 471 MHz, respectively. The abbreviations used for peak multiplicities are as follows: s: singlet, d: doublet, t: triplet, q: quadruplet, dd = doublet of doublet, br = broad, and m: multiplet. The coupling constants J are in Hz, and chemical shifts are given in ppm and calibrated with CDCl_3_ or CD_3_OD (residual solvent signals). ^19^F NMR chemical shifts (δ) were determined relative to CFCl_3_ as an internal standard (^19^F, δ = 0.0 ppm). The LC–MS analyses were performed on a Waters Acquity UPLC H-ClassXevo G2-XS Q-TOF. High resolution mass spectra (HRMS) were recorded using a Waters Q-TOF microspectrometer using electrospray ionization (ESI). Pre-conditioned ABX Sep-Pak^®^ Light QMA cartridges were used as received. Waters Sep-Pak^®^ tC18 Plus Long Environmental cartridges were used after pre-conditioning with EtOH (10 mL) and water (20 mL).

#### 3.1.2. 3-(Prop-2-yn-1-yl)-1,2-oxathiane 2,2-dioxide **3**

1,4-Butanesultone **2** (2 mL, 21.2 mmol) was placed in anhydrous and degassed THF (8 mL) in a three-neck round-bottom flask and under inert atmosphere. The resulting solution was cooled to −78 °C. Then, n-Butyllithium (11 M in hexane, 3 mL, 33.0 mmol) was added dropwise at −78 °C for 30 min. In parallel, 3-bromopropyne (2 mL, 80% in toluene, 21.5 mmol) was added dropwise for 30 min, and then the mixture was stirred for 20 min at −78 °C. The reaction was quenched by the addition of a mixture of H_2_O/AcOEt (60 mL, *v:v* 1/1) at −78 °C. After extraction with AcOEt (3 × 10 mL), the organic phase was washed with water (2 × 10 mL), dried over MgSO_4_, filtered, and concentrated under reduced pressure. The residue was purified through silica flash chromatography, using n-pentane/Et_2_O (70/30) as an eluent to obtain product **3** as a white powder (1.48 g, 40%). R_f_: 0.38 (n-pentane/AcOEt 3/2); Mp: 58–60 °C (after sublimation); ^1^H NMR (500 MHz, CDCl_3_): δ 4.59–4.45 (m, 2H), 3.28–3.19 (m, 1H), 3.03–2.93 (m, 1H), 2.59–2.53 (m, 1H), 2.53–2.46 (m, 1H), 2.13–2.10 (m, 1H), 2.10–2.01 (m, 1H), 1.95–1.87 (m, 2H); ^13^C NMR (126 MHz, CDCl3): δ 78.1, 74.2, 74.2, 72.0, 57.6, 27.6, 23.6, 18.5; HRMS (ESI^−^): calculated for C_7_H_9_O_3_S: 173.0272 [M − H]^−^, found: 173.0253.

#### 3.1.3. 7-(Fluoro)hept-1-yne-4-sulfonic Acid **4**

A mixture of sultone **3** (5.0 mg, 28.5 µmol) and cesium fluoride (4.4 mg, 29.0 µmol) in acetonitrile (1 mL) was refluxed for 6 h, then cooled to 20 °C. After concentration under reduced pressure at 40 °C, the residue was solubilized in water (1 mL), and the resulting solution was passed onto a Sep-Pak^®^ Light QMA. After QMA elution with acetonitrile (3 × 0.5 mL), the combined eluates were concentrated under reduced pressure at 40 °C to afford title product **4** as a white powder (9.2 mg, 99%), which was used directly without further purification. R_f_: 0.95 (CH_3_CN/TFA 98/02); Mp: > 190 °C (decomposition); ^1^H NMR (500 MHz, CD_3_OD): δ 4.50–4.28 (m, 2H), 2.88–2.70 (m, 2H), 2.45–2.34 (m, 1H), 2.32 (t, J = 2.8 Hz, 1H), 2.00–1.87 (m, 4H); ^13^C NMR (126 MHz, CD_3_OD): δ 86.1, 83.5, 81.5, 71.0, 58.0, 25.9, 20.6; ^19^F NMR (471 MHz, CDCl_3_): δ–215.3; HRMS (ESI^-^): calculated for C_7_H_10_FO_3_S: 193.0335 [M − H]^−^, found: 193.0342.

#### 3.1.4. 4-(Azidomethyl)benzonitrile **6**

In a 50 mL round-bottom flask, 4-(bromomethyl)benzonitrile **5** (5.1 g, 26.2 mmol), sodium azide (8.5 g, 130.6 mmol), and potassium iodide (2.1 g, 12.7 mmol) were stirred in acetone (15 mL) at 27 °C for 15 h. After filtration, purification on silica using DCM as the eluent afforded product **6** (4.0 g, 97%) as a light-yellow oil. R_f_: 0.34 (n-pentane/DCM 80/20); ^1^H NMR (500 MHz, CDCl_3_): δ 7.65–7.52 (m, 2H), 7.38 (d, J = 8.1 Hz, 2H), 4.39 (s, 2H); ^13^C NMR (126 MHz, CDCl_3_): δ 140.6, 132.3, 128.3, 118.2, 111.6, 53.6; HRMS (ESI^+^): calculated for C_8_H_7_N_4_: 159.0671 [M + H]^+^, found: 158.9929.

#### 3.1.5. 3-(4-(Azidomethyl)phenyl)-6-methyl-1,2,4,5-tetrazine **7**

In a 250 mL round-bottom flask, nitrile **6** (2.0 g, 12.7 mmol), 3-mercaptopropionic acid (1 mL, 12.6 mmol), acetonitrile (9 mL, 179.6 mmol), hydrazine monohydrate (50%, 12 mL, 202.3 mmol), and ethanol (10 mL) were stirred at 45 °C for 24 h. After concentration under reduced pressure at 40 °C, sodium nitrite (6.9 g, 98.7 mmol) in water (20 mL) was added to the residue. Then, a solution of formic acid (10 mL, 265.0 mmol) in water (10 mL) was carefully added dropwise over 10 min at −5 °C (leading to a pH of 4–5). The final mixture was stirred at −5 °C for 5 min. After extraction with diethyl ether (3 × 10 mL), the combined organic phases were washed with sodium thiosulfate (5%, 2 × 5 mL), dried over MgSO_4_, filtered off, and concentrated under reduced pressure at 40 °C. The residue was purified with flash chromatography, using n-pentane/DCM (from 100/0 to 0/100) as the eluent to yield tetrazine **7** as a red powder (1.1 g, 48%). R_f_: 0.32 (n-pentane/DCM 30/70); Mp: 108–110 °C; ^1^H NMR (500 MHz, CDCl_3_): δ 8.67–8.53 (m, 2H), 7.57–7.50 (m, 2H), 4.48 (s, 2H), 3.11 (s, 3H); ^13^C NMR (126 MHz, CDCl_3_): δ 167.5, 163.9, 140.2, 131.9, 129.0, 128.5, 54.5, 21.3; HRMS (ESI^+^): calculated for C_10_H_10_N_7_: 228.0998 [M + H]^+^, found: 228.0995.

#### 3.1.6. 3-((1-(4-(6-Methyl-1,2,4,5-tetrazin-3-yl)benzyl)-1H-1,2,3-triazol-4-yl)methyl)-1,2-oxathiane 2,2-dioxide **8**

In a 250 mL round-bottom flask under nitrogen, a mixture of tetrazine **7** (1.5 g, 6.5 mmol), sultone **3** (1.1 g, 6.5 mmol), copper (II) acetate (0.1 g, 0.6 mmol), and sodium ascorbate (0.1 g, 0.7 mmol) in acetone (6 mL) was refluxed for 2 h; then, it was concentrated under reduced pressure at 30 °C. The residue was diluted in AcOEt (10 mL), and the resulting mixture was filtered through Celite^®^ 545 (10 g). After washing the Celite with AcOEt (5 × 20 mL), the combined eluates were concentrated under reduced pressure at 30 °C. The residue was purified with flash chromatography using n-pentane/AcOEt (from 50/01 to 0/100) as the eluent to yield tetrazine sultone **8** as a red powder (1.3 g, 49%). R_f_: 0.33 (AcOEt); Mp: 130 °C; ^1^H NMR (500 MHz, CDCl_3_): δ 8.59–8.55 (m, 2H), 7.50 (s, 1H), 7.46–7.42 (m, 2H), 5.62 (s, 2H), 4.59–4.50 (m, 1H), 4.50–4.42 (m, 1H), 3.53–3.46 (m, 1H), 3.44–3.38 (m, 1H), 3.09 (s, 3H), 3.03 (dd, J = 15.2, 8.1 Hz, 1H), 2.32–2.25 (m, 1H), 2.07–1.95 (m, 1H), 1.95–1.81 (m, 2H); ^13^C NMR (126 MHz, CDCl_3_): δ 167.7, 163.7, 139.1, 132.4, 128.8, 128.8, 123.0, 122.8, 74.1, 59.2, 53.9, 28.7, 25.2, 24.0, 21.4; HRMS (ESI^+^): calculated for C_17_H_20_N_7_O_3_S: 402.1348 [M + H]^+^, found: 402.1345.

#### 3.1.7. 5-(Fluoro)-1-(1-(4-(6-methyl-1,2,4,5-tetrazin-3-yl)benzyl)-1H-1,2,3-triazol-4-yl)pentane-2-sulfonic Acid **1**

In a 2 mL conical Reactivial^®^ (from Thermo Fisher), a solution of sodium ascorbate (30.5 mg, 0.1 mmol) in water (150 µL) was added to a solution of copper (II) acetate (8.5 mg, 46.5 µmol) in water (100 µL). The mixture was stirred under a continuous flow of nitrogen for 1 min, and was then added to the vial containing sulfonate **4** (9.3 mg, 28.5 µmol). After stirring for 1 min, a solution of tetrazine **8** (5.2 mg, 22.8 µmol) in acetone (0.7 mL) was added under nitrogen flow. After refluxing for 30 min, a solution of sodium nitrite (30.0 mg, 0.4 mmol) in water (0.1 mL) was added dropwise over 5 min; then, formic acid (20 µL, 0.5 mmol) diluted in water (0.1 mL) was added dropwise over 5 min. The final mixture was stirred at 0 °C for 5 min. Water (2 × 5 mL) was added, and the mixture was passed through a pre-conditioned Sep-Pak^®^ tC18 Plus Long Environmental. The Sep-Pak was washed with water (10 mL) and dried. After elution with ethanol (5 mL) and then concentration under reduced pressure at 40 °C of the ethanolic solution, the residue was purified by semi-preparative HPLC [Phenomenex Gemini^®^ C18, 5 µm, 250 × 10 mm, with phosphate buffer solution at pH 2.1/EtOH 72/28, 4.0 mL/min, λ = 254 nm]. The fraction collected (t_R_ = 28–29 min) was concentrated under reduced pressure at 20 °C, diluted in water, and passed through a pre-conditioned Sep-Pak^®^ tC18 Plus Long Environmental. After washing with water (10 mL) and elution with ethanol (1 mL), the ethanolic solution was concentrated under reduced pressure to yield fluorotetrazine **1** as a red powder (11.0 mg, 92%). R_f_: 0.33 (CH_3_OH); Mp: > 190 °C (decomposition); ^1^H NMR (500 MHz, CDCl_3_): δ 8.60 (d, J = 8.0 Hz, 2H), 7.69 (s, 1H), 7.43 (d, J = 8.0 Hz, 2H), 5.60 (s, 2H), 4.44–4.24 (m, 2H), 3.44–3.36 (m, 1H), 3.13–3.01 (s, 3H), 3.01–2.92 (m, 2H), 2.49 (s, 1H), 2.05–1.82 (m, 2H), 1.76–1.64 (m, 2H); ^13^C NMR (126 MHz, CDCl_3_): δ 167.7, 163.7, 139.1, 132.4, 128.8, 128.8, 123.1, 122.8, 74.1, 59.2, 53.9, 28.7, 25.2, 24.0, 21.4; ^19^F NMR (471 MHz, CDCl_3_): −218.7; HRMS (ESI^-^): calculated for C_17_H_19_FN_7_O_3_S: 420.1254 [M − H]^−^, found: 420.1256.

### 3.2. Automated Radiosynthesis of [^18^F]***1***

#### 3.2.1. General

[^18^F]Fluoride was produced according to the ^18^O[p,n]^18^F nuclear reaction by irradiation of ^18^O-enriched water (97%, Eurisotop) with a IBA Cyclone^®^ 18/9 cyclotron. A TRACERlab FX NPro synthesis module (GE Medical Systems) was used for the automated radiosynthesis of [^18^F]1. The purification and isolation of [^18^F]1 were performed using semi-preparative HPLC [Phenomenex Gemini^®^ C18 column, 5 µm, 250 × 10 mm, with phosphate buffer (pH 2.1)/EtOH (72/28) as the eluent, and a 3.3 mL/min flow rate, λ = 254 nm]. Analytical HPLC for quality control and stability studies was performed using a Waters system [C18 Gemini column, 5 µm, 4.6 × 250 mm, 110 A; NH_4_OAc (10 mM)/ACN (55/45) as the eluent; 1 mL/min flow-rate]. The radioTLC analyses were carried out using Merck 60F_254_ silica gel deposited on a glass plate, with CH_3_CN/H_2_O containing 0.1% TFA (98/02) as the eluent. RadioTLCs were measured on an Elysia Raytest Rita Star 2018203 plate reader. The identity of the radiolabeled compounds was determined via HPLC and TLC analyses by comparison and co-elution with the non-radiolabeled analogue. Pre-conditioned ABX Sep-Pak^®^ Light QMA cartridges were used as received. Waters Sep-Pak^®^ tC18 Plus Long Environmental cartridges were used after pre-conditioning with EtOH (10 mL) and water (20 mL).

#### 3.2.2. 5-([^18^F]Fluoro)-1-(1-(4-(6-methyl-1,2,4,5-tetrazin-3-yl)benzyl)-1H-1,2,3-triazol-4-yl)pentane-2-sulfonic Acid [^18^F]**1**

A cyclotron-produced solution of [^18^F]fluoride in ^18^O-enriched water was passed on a pre-conditioned Sep-Pak^®^ Light QMA. The [^18^F]fluoride was eluted with a solution of potassium carbonate (0.5 mg) and Kryptofix K_222_ (10.5 mg) in water/acetonitrile (500 μL/1 mL). The resulting [^18^F]KF/K_2_CO_3_/K_222_ solution was concentrated under reduced pressure (0.04 kPa) at 65 °C for 6 min, then at 95 °C for 3 min. After cooling to 70 °C, a solution of propargylic sultone **3** (5.0 mg) in CH_3_CN (1.6 mL) was added. The mixture was heated at 105 °C for 15 min, cooled to 50 °C, and concentrated under reduced pressure (0.04 kPa) for 4 min to yield crude propargylic [^18^F]fluorosulfonate [^18^F]**4**. Acetone (1.6 mL) was added to the residue [^18^F]**4**, and the resulting mixture was passed onto a Sep-Pak^®^ Light QMA. The eluate containing [^18^F]**4** was concentrated under reduced pressure (0.04 kPa) at 50 °C for 4 min. A second addition of acetone (1.6 mL) into the radiofluorination reactor was realized, and the resulting solution was passed onto a Sep-Pak^®^ Light QMA. The eluate was added to the previous pre-purified [^18^F]**4** fraction and concentrated at 40 °C under reduced pressure (0.04 kPa) for 30 s, in order to recover about 350 μL of solution. In parallel, an aqueous sodium ascorbate solution (30.0 mg in 150 μL of H_2_O) was added to an aqueous copper acetate solution (8.4 mg in 150 μL of H_2_O). The resulting mixture was bubbled with helium flow for 20 s, then added to pre-purified [^18^F]**4**. After stirring for 20 s, sublimated azidotetrazine **7** (5.2 mg) in acetone (350 μL) was added. The final mixture was heated at 60 °C for 25 min, and cooled to 30 °C. A solution of sodium nitrite (30.5 mg) in H_2_O (300 μL) was introduced, and immediately after formic acid (100 μL). The mixture was stirred at 25–30 °C under helium for 5 min, then transferred into a large-volume vial for dilution. Dilution was performed by the addition of water (2 × 5 mL). The resulting mixture was passed onto a pre-conditioned Sep-Pak^®^ tC18 Plus Long Environmental. After washing with water (10 mL), the Sep-Pak^®^ tC18 was eluted with EtOH (1.5 mL). The ethanolic solution was diluted in phosphate buffer (3.5 mL, pH 2.1), and injected into semi-preparative reverse phase HPLC. The [^18^F]fluorosulfotetrazine [^18^F]**1** was collected in a 72/28 mixture of phosphate buffer pH 2.1/EtOH (5 mL) over 1.5 min, from about 18 to 19.5 min after injection, then formulated by the addition of sodium bicarbonate in physiological serum (Lavoisier NaHCO_3_, 1.4%, 0.8 mL,) and Baxter NaCl Viaflo (0.9%, 9.2 mL). Using the automated process, the [^18^F]fluorosulfotetrazine [^18^F]**1** (86 mCi ≈ 3.2 GBq) was obtained in a formulated solution ready for injection (15 mL total volume) at a 29–35% decay-corrected yield after 90–95 min of total time synthesis, and a molar activity of about 165 MBq/μmol (4.4 mCi/μmol), starting from about 18 GBq (485 mCi) of cyclotron-produced [^18^F]fluoride. Analytical HPLC of aliquots of the formulated solution displayed >97% radiochemical purity and >91% chemical purity.

### 3.3. Radiochemical and Chemical Purities, and Molar Activity of [^18^F]***1***

Aliquots of the formulated solution of [^18^F]**1** were used to establish the chemical and radio-chemical purities, and molar activity. The determination was carried out using analytical HPLC and radio-TLC.

### 3.4. LogP and LogD_7.4_ Calculations

Octanol (1 mL) and water or PBS buffer (1 mL) were placed in a hemolysis tube, and mixed together for 20 min at room temperature before the addition of formulated [^18^F]**1** (4 µL, ≈0.185 MBq). Then, the tubes were vigorously shaken for 40 min. After centrifugation (4024× *g*, 5 min), samples (3 × 100 µL) from each phase were collected, and the radioactivity was measured in a γ-counter (Perkin wizard 2 gamma detector series 2470). The experiment was carried out twice in triplicate.

### 3.5. In Vitro Stability Studies

Aliquots of formulated [^18^F]**1** (100 μL) were diluted, either in the formulation medium or in PBS buffer, pH 7.4. The resulting mixtures were stirred at 37 °C. Analytical radioHPLC analyses were performed every 3 h for 9 h.

### 3.6. Animal Studies

#### 3.6.1. General Considerations

The animal investigations were performed under the current European directive (2010/63/EU), as incorporated in national legislation and in authorized laboratories (GIP Cyceron; E14118001). The experimental procedures were preliminarily approved from the regional committee on animal ethics (approval #3247). Healthy male SWISS mice were obtained from an in-house breeding stock at the “Centre Universitaire de Ressources Biologiques” (CURB; A14118015). All of the animals were housed in groups of 2 or more, with 12/12 h light-dark cycles, and with food and water ad libitum. The animals were maintained under isoflurane anesthesia throughout all procedures (induction 5%, maintenance around 2.5%, with 70% N_2_O/30% O_2_), and their body temperatures were maintained close to 37.5 °C using a feedback-controlled system (Minerve Veterinary Equipment, France) during experimentation. A catheter was inserted into the tail vein without surgery (Insyte™ Autoguard™, BD Medical, USA) for intravenous administration. Euthanasia of the animals was performed at the end of the protocol, using an isoflurane (5%) overdose.

#### 3.6.2. PET Imaging Experiments

Imaging studies were performed on a Inveon μPET/CT scanner (Siemens Healthcare Molecular Imaging). The respiratory rate was monitored during imaging sessions. List-mode PET data were acquired for 60 min, and this was initiated as soon as the formulated [^18^F]**1** (~5.6 MBq/100 μL) was injected. The PET images were reconstructed using an iterative OSEM3D/MAP algorithm. Dead-time, random, scatter, as well as attenuation correction (based on CT) were applied. The image analysis was performed with P-Mod 3.7 software (P-MOD Technologies). Briefly, the PET and CT images were co-registered. Volumes of interest (VOIs) were semi-automatically delimitated for the following organs: bladder, bone, brain, heart, kidney, liver, lung, and muscle, if available. Time-activity curves (TACs) were extracted from PET images, with the data expressed as standardized uptake value (SUVMean). SUV refers to the ratio of tissue radioactivity concentration at time t and administered dose at the time of [^18^F]1 injection, divided by body weight.

#### 3.6.3. In Vivo Stability Studies

Blood (≈1 mL) was sampled using intra-cardiac puncture, 30 min post-injection (29 MBq/100 μL); it was then heparinized and centrifuged (4024× *g*, 5 min, 4 °C). Plasma was collected, mixed with one equivalent volume of acetonitrile, and centrifuged again (4024× *g*, 10 min, 4 °C; >90% extraction yield). The supernatant was filtered through a 0.45 μm PVDF, and then injected into analytical HPLC.

## 4. Conclusions

In this study, original fluorotetrazine **1**, lead compound of a new class of tetrazines bearing a sulfo group and a triazole ring, was designed, synthesized, and radiolabeled with fluorine-18. Next, the in vivo behavior of the radioactive [^18^F]**1** was examined. The synthesis of **1** was accomplished efficiently using a CuAAC reaction between the novel azidotetrazine **7** and fluoroheptynylsulfonic acid **4**. The tetrazine **1** demonstrated high reactivity towards the TCO reagent, rendering promising further extensions to the radiolabelling of biologics. The optimized radiosynthesis of [^18^F]**1** involved the ring opening of propargylbutanesultone **2** with [^18^F]fluoride to afford [^18^F]fluoroheptynylsulfonic acid [^18^F]**4**, which reacted with the azidotetrazine **7** via the CuAAC reaction to provide [^18^F]**1** in acceptable radiochemical yields, with high chemical and radiochemical purities. As expected, [^18^F]**1** displayed hydrophilic properties, as shown by the LogP and LogD_7.4_ values, but also by the rapid and predominant renal and urinary excretion revealed by in vivo PET imaging. We also observed a fast clearance of radioactivity from tissues, an absence of non-specific uptake, and a high metabolic stability, making [^18^F]**1** a suitable bioorthogonal reagent. Its evaluation for peptides radiolabeling as well as for pre-targeting applications is currently under investigation. This first fluorosulfotetrazine **1** also opens the way to the development of original analogues, with both increased hydrophilicity and reactivity in IEDDA reactions, in order to improve imaging. Chemical modifications of **1**, such as the replacement of the methyl group by a pyridine substituent, are currently underway.

## Data Availability

Data is contained within the article and supplementary material.

## Supplementary Materials

The following supporting information can be downloaded at: https://www.mdpi.com/article/10.3390/ph16050636/s1, Figure S1: ^1^H NMR spectrum of compound **3**; Figure S2: ^13^C NMR spectrum of compound **3**; Figure S3: MS and HRMS analysis of compound **3**; Figure S4: ^1^H NMR spectrum of compound **4**; Figure S5: ^13^C NMR spectrum of compound **4**; Figure S6: ^19^F NMR spectrum of compound **4**; Figure S7: MS and HRMS analysis of compound **4**; Figure S8: ^1^H NMR spectrum of compound **6**; Figure S9: ^13^C NMR spectrum of compound **6**; Figure S10: MS and HRMS analysis of compound **6**; Figure S11: ^1^H NMR spectrum of compound **7**; Figure S12: ^13^C NMR spectrum of compound **7**; Figure S13: MS and HRMS analysis of compound **7**; Figure S14: ^1^H NMR spectrum of compound **8**; Figure S15: ^13^C NMR spectrum of compound **8**; Figure S16: MS and HRMS analysis of compound **8**; Figure S17: ^1^H NMR spectrum of compound **1**; Figure S18: ^13^C NMR spectrum of compound **1**; Figure S19: ^19^F NMR spectrum of compound **1**; Figure S20: MS and HRMS analysis of compound **1**; Figure S21: MS analysis of compound **1**; Figure S22: RadioTLC analysis of crude compound [^18^F]**4**; Figure S23: HPLC analysis of crude compound [^18^F]**4**.
