# Supplementary material for: Synthesis, Fluorine-18 Radiolabeling, and In Vivo PET Imaging of a Hydrophilic Fluorosulfotetrazine
Source: Pharmaceuticals (Basel). 2023 Apr 22;16(5):636. doi: 10.3390/ph16050636 (PMC10221973; doi:10.3390/ph16050636)
Supplement: Supplementary file 1 [file pharmaceuticals-16-00636-s001.zip › pharmaceuticals-2329865-supplementary.pdf]

Synthesis, Fluorine-18 Radiolabeling, and In Vivo PET Imaging of a Hydrophilic Fluorosulfotetrazine

Jason Beaufrez [1,2], Stéphane Guillouet [1], Yann Seimbille [2] and Cécile Perrio [1,*]

[1] UAR 3408, CNRS, CEA, Unicaen, Cyceron, Bd Henri Becquerel, 14074 Caen, France
[2] Department of Radiology & Nuclear Medicine, Erasmus MC, University Medical Center Rotterdam, Wytemaweg 80, 3015 CN Rotterdam, The Netherlands
* Correspondence: perrio@cyceron.fr

Figure S1. ^{1}H NMR spectrum of compound **3** **p.S2**
Figure S2. ^{13}C NMR spectrum of compound **3** **p.S2**
Figure S3. MS and HRMS analysis of compound **3** **p.S3**
Figure S4. ^{1}H NMR spectrum of compound **4** **p.S3**
Figure S5. ^{13}C NMR spectrum of compound **4** **p.S4**
Figure S6. ^{19}F NMR spectrum of compound **4** **p.S4**
Figure S7. MS and HRMS analysis of compound **4** **p.S5**
Figure S8. ^{1}H NMR spectrum of compound **6** **p.S5**
Figure S9. ^{13}C NMR spectrum of compound **6** **p.S6**
Figure S10. MS and HRMS analysis of compound **6** **p.S6**
Figure S11. ^{1}H NMR spectrum of compound **7** **p.S7**
Figure S12. ^{13}C NMR spectrum of compound **7** **p.S7**
Figure S13. MS and HRMS analysis of compound **7** **p.S8**
Figure S14. ^{1}H NMR spectrum of compound **8** **p.S8**
Figure S15. ^{13}C NMR spectrum of compound **8** **p.S9**
Figure S16. MS and HRMS analysis of compound **8** **p.S9**
Figure S17. ^{1}H NMR spectrum of compound **1** **p.S10**
Figure S18. ^{13}C NMR spectrum of compound **1** **p.S10**
Figure S19. ^{19}F NMR spectrum of compound **1** **p.S11**
Figure S20. MS and HRMS analysis of compound **1** **p.S11**
Figure S21. MS analysis of compound **10** **p.S12**
Figure S22. RadioTLC analysis of crude compound [^{18}F]**4** **p.S12**
Figure S23. HPLC analysis of crude compound [^{18}F]**4** **p.S13**

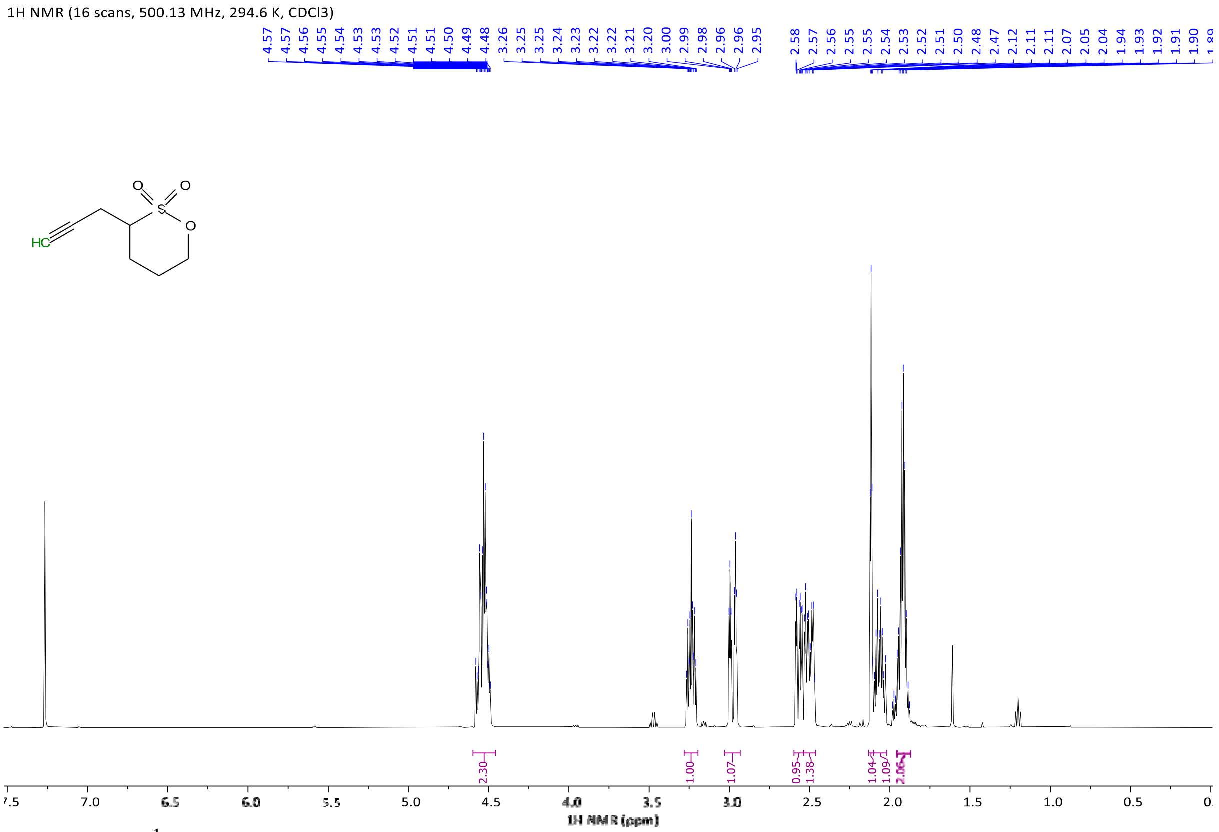

Figure S1. ^{1}H NMR spectrum of compound **3**.

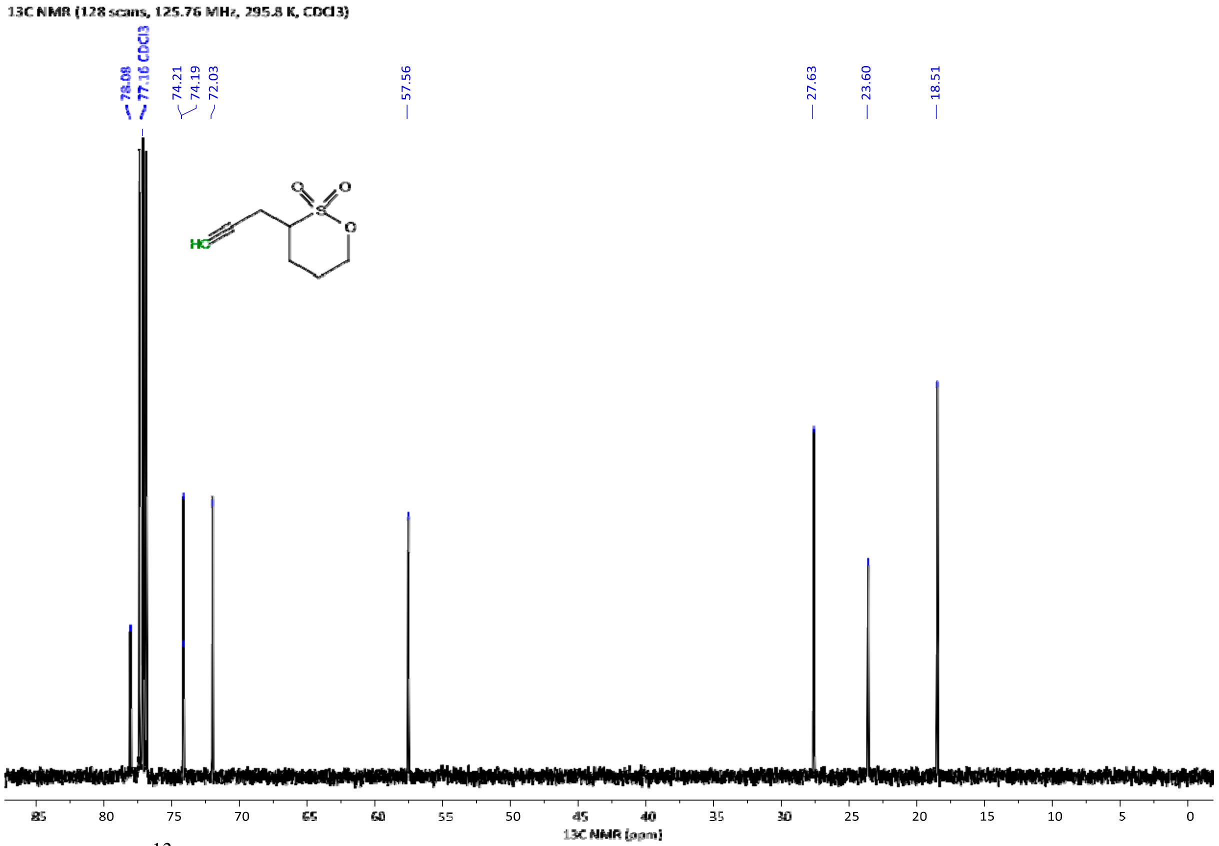

Figure S2. ^{13}C NMR spectrum of compound **3**.

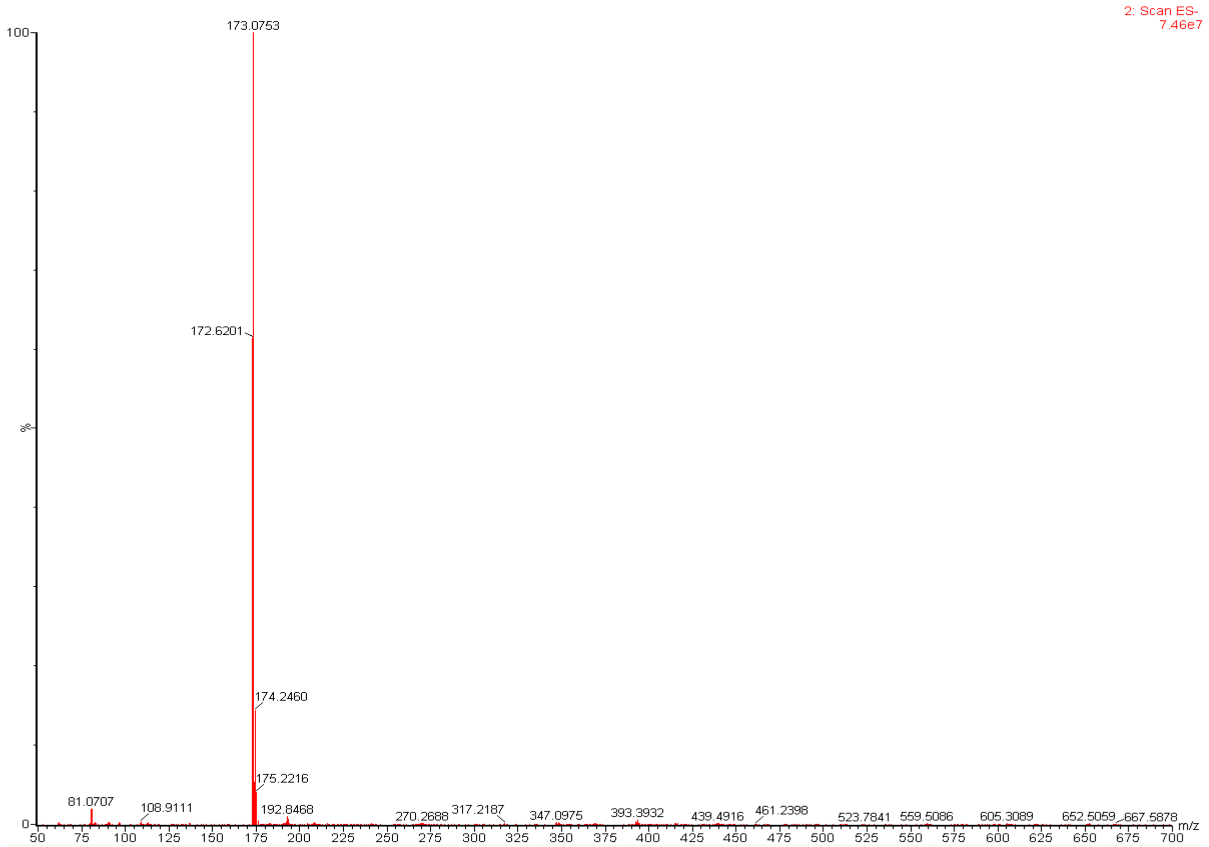

Mass	Calc. Mass	mDa	PPM	DBE	i-FIT	Norm	Conf(%)	Formula
173.0253	173.0272	48.1	277.9	3.5	109.0	6.628	0.13	C7 H9 O3 S

Figure S3. MS and HRMS analysis of compound **3**.

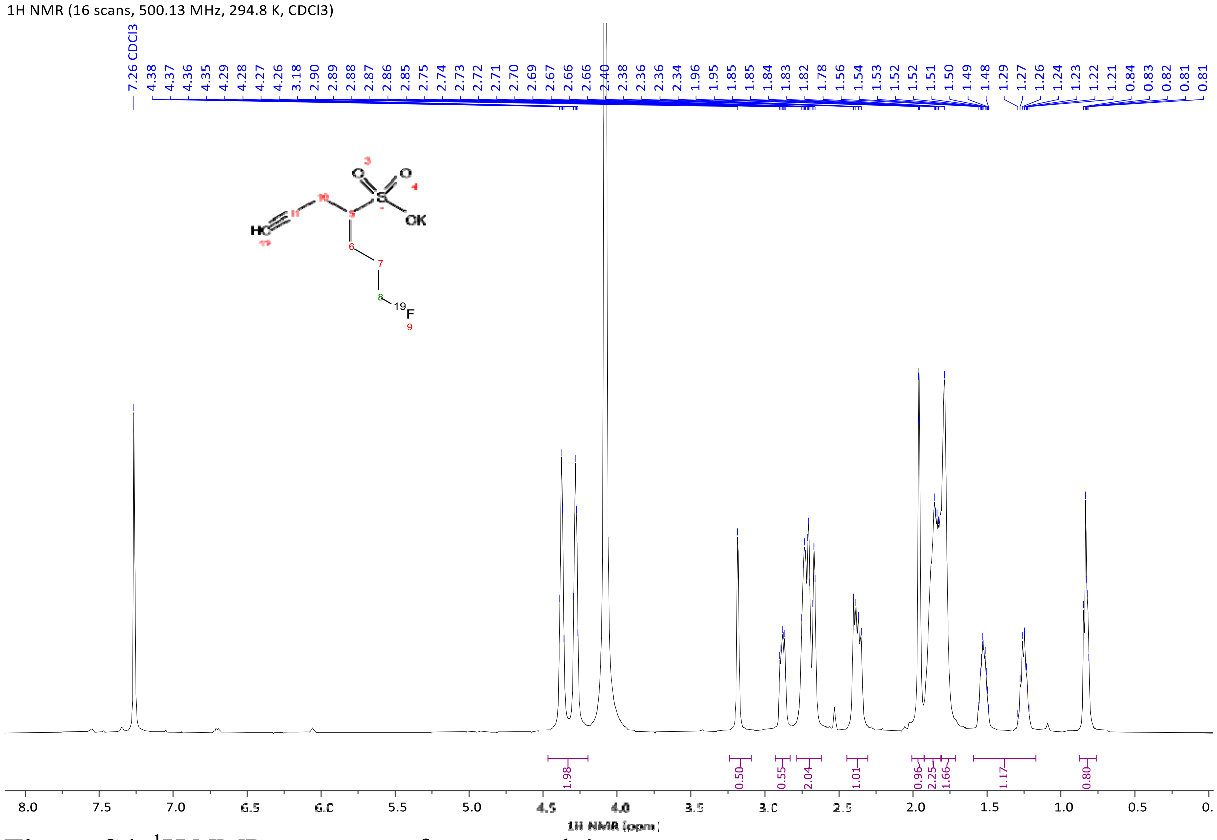

Figure S4. ^{1}H NMR spectrum of compound **4**.

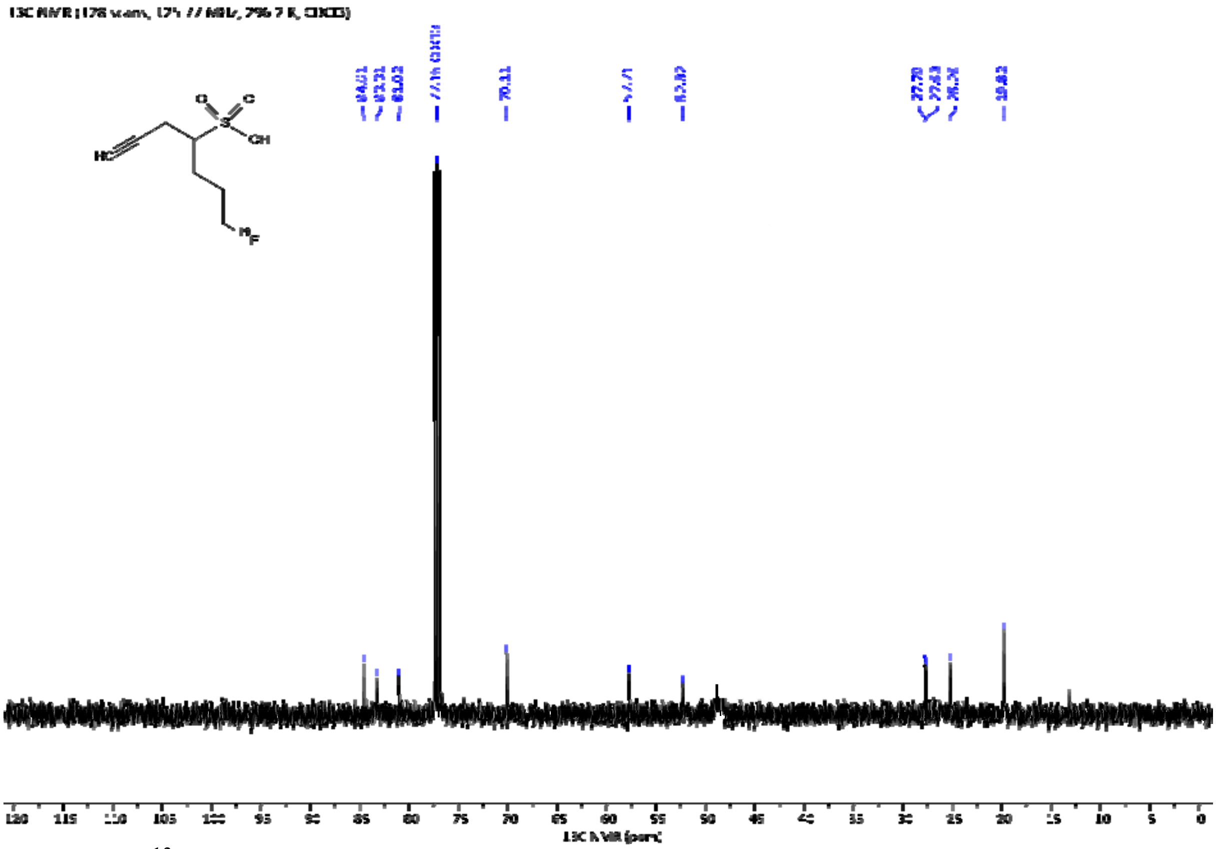

Figure S5. ^{13}C NMR spectrum of compound **4**.

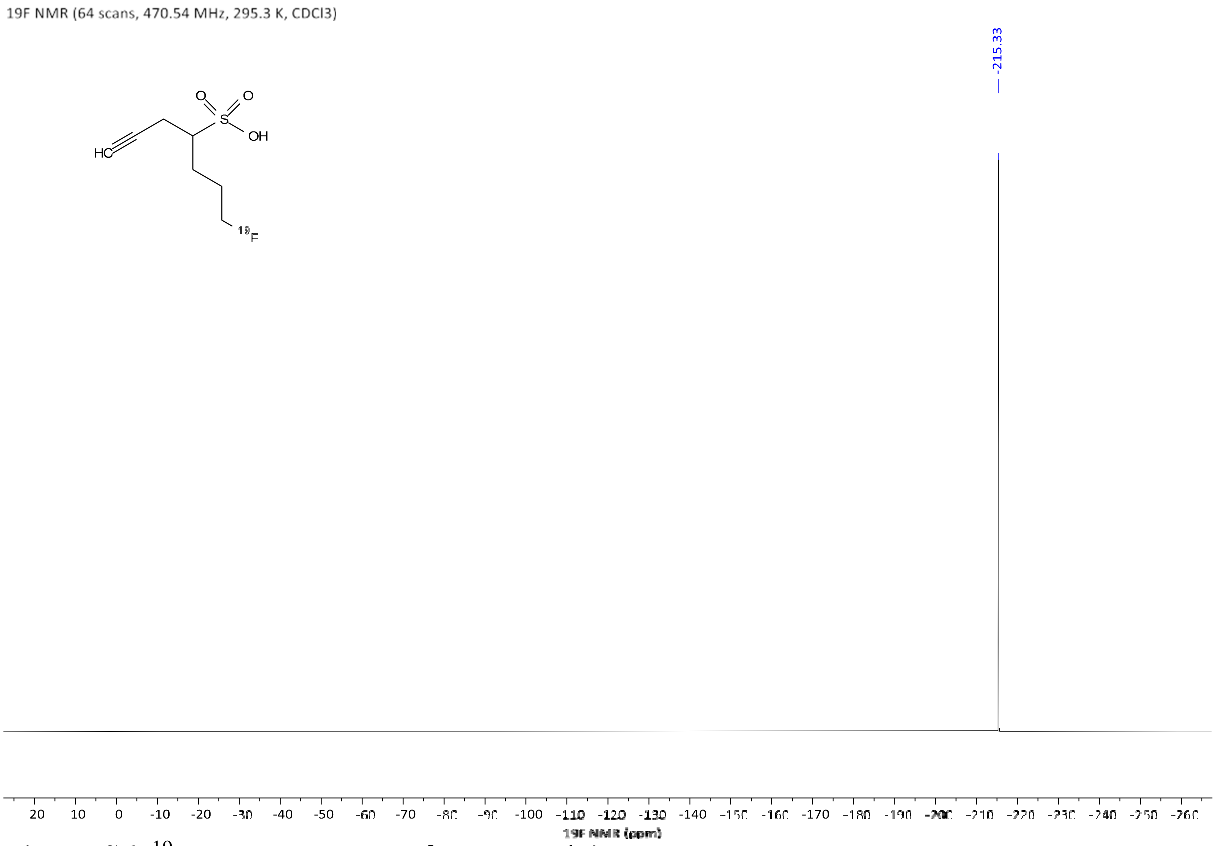

Figure S6. ^{19}F NMR spectrum of compound **4**.

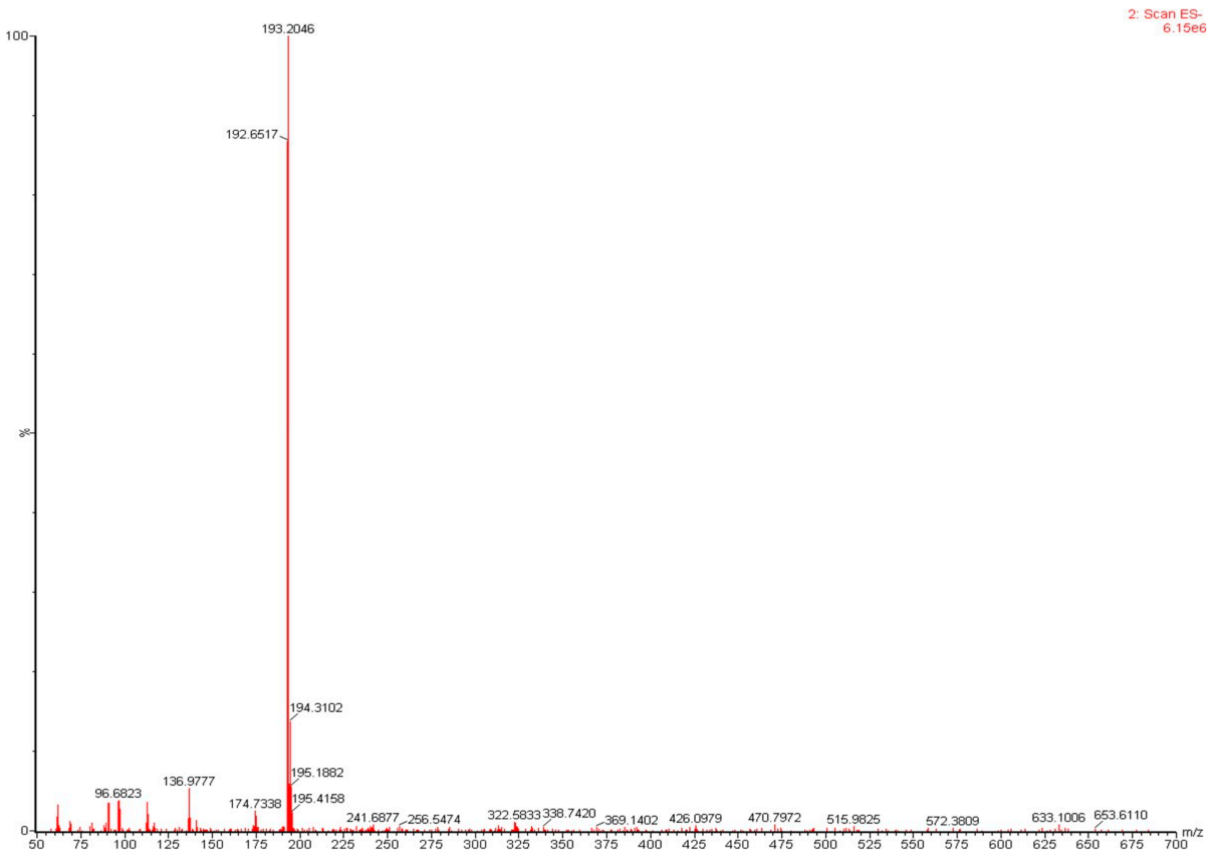

Mass	Calc. Mass	mDa	PPM	DBE	i-FIT	Norm	Conf(%)	Formula
193.0342	193.0335	171.1	885.6	2.5	65.1	15.131	0.00	C7 H10 O3 F S

Figure S7. MS and HRMS analysis of compound **4**.

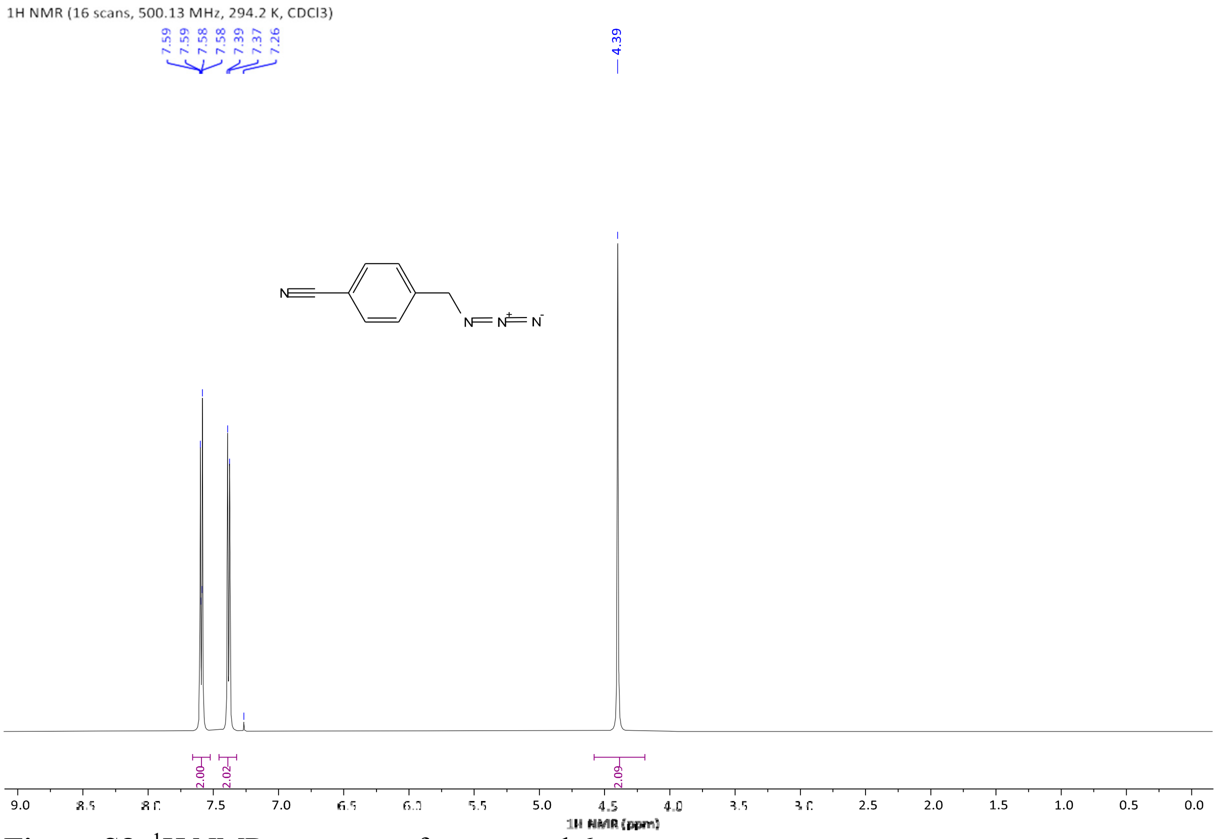

Figure S8. ^{1}H NMR spectrum of compound **6**.

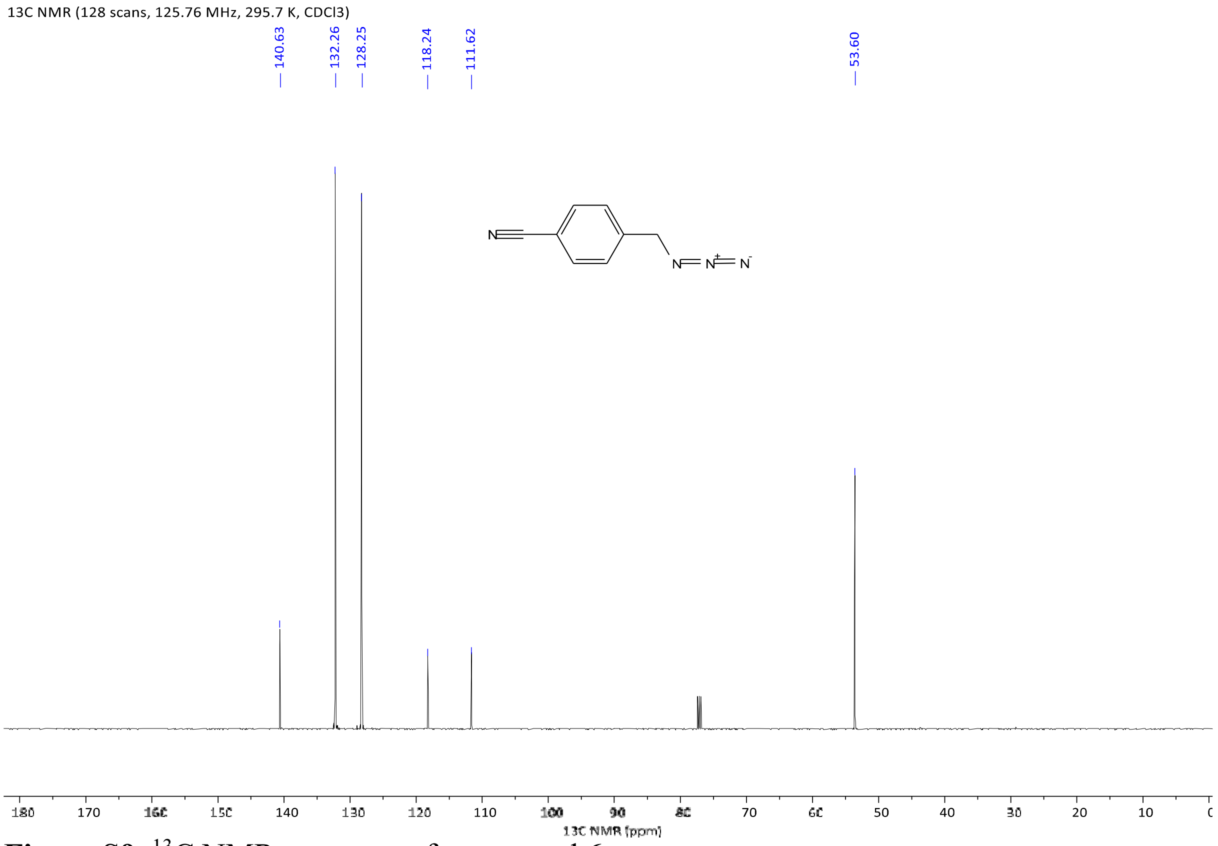

Figure S9. ^{13}C NMR spectrum of compound **6**.

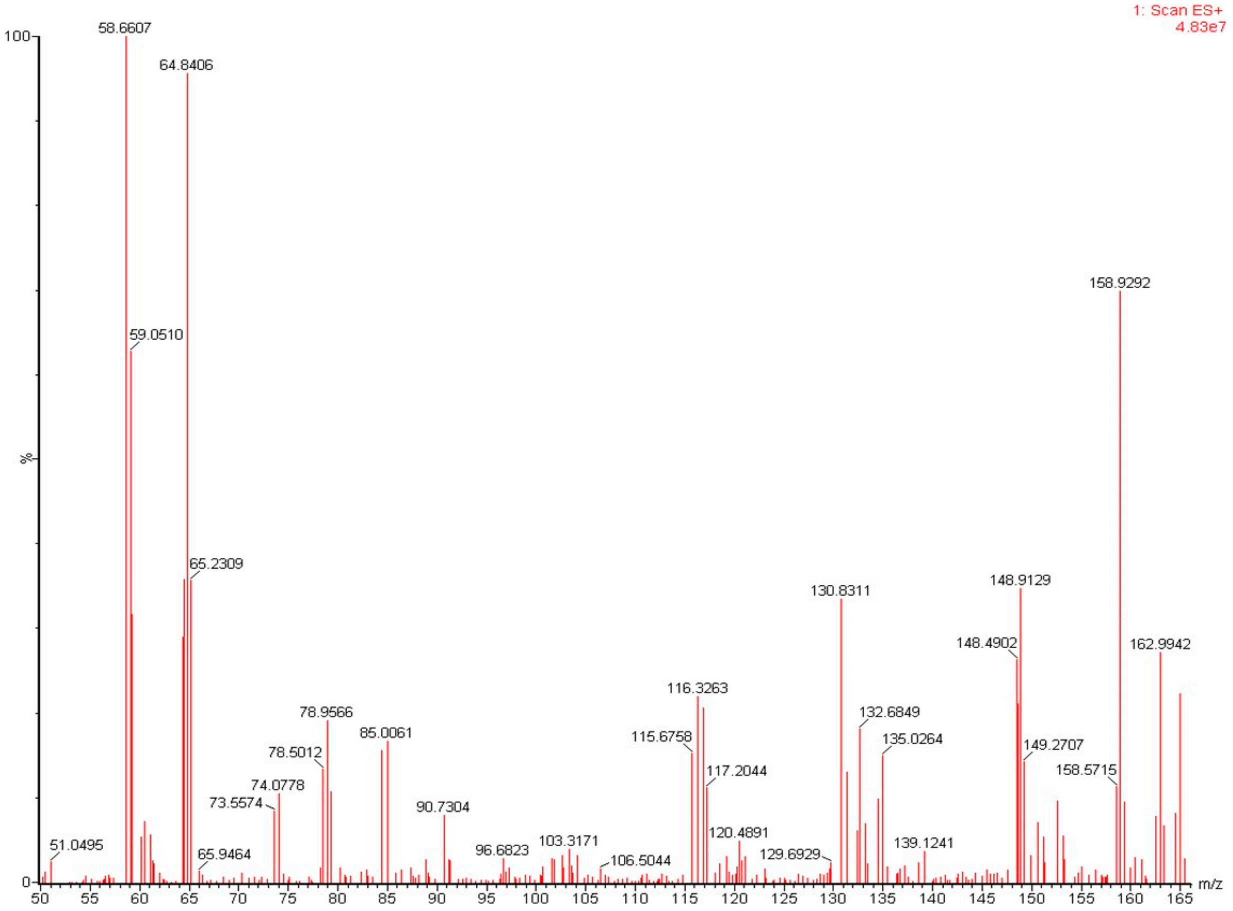

Mass	Calc. Mass	mDa	PPM	DBE	i-FIT	Norm	Conf(%)	Formula
158.9929	159.0671	-74.2	-466.7	7.5	100.0	13.900	0.00	C8 H7 N4

Figure S10. MS and HRMS analysis of compound **6**.

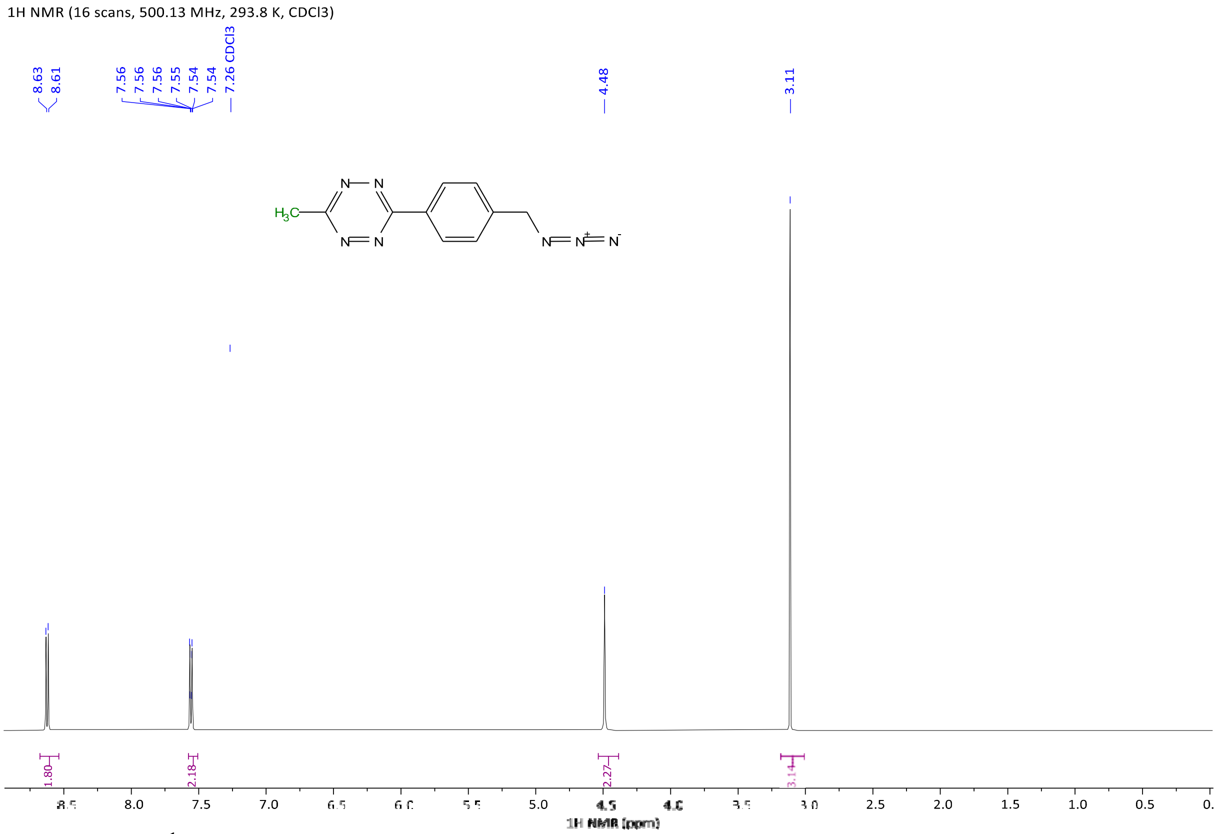

Figure S11. ^{1}H NMR spectrum of compound **7**.

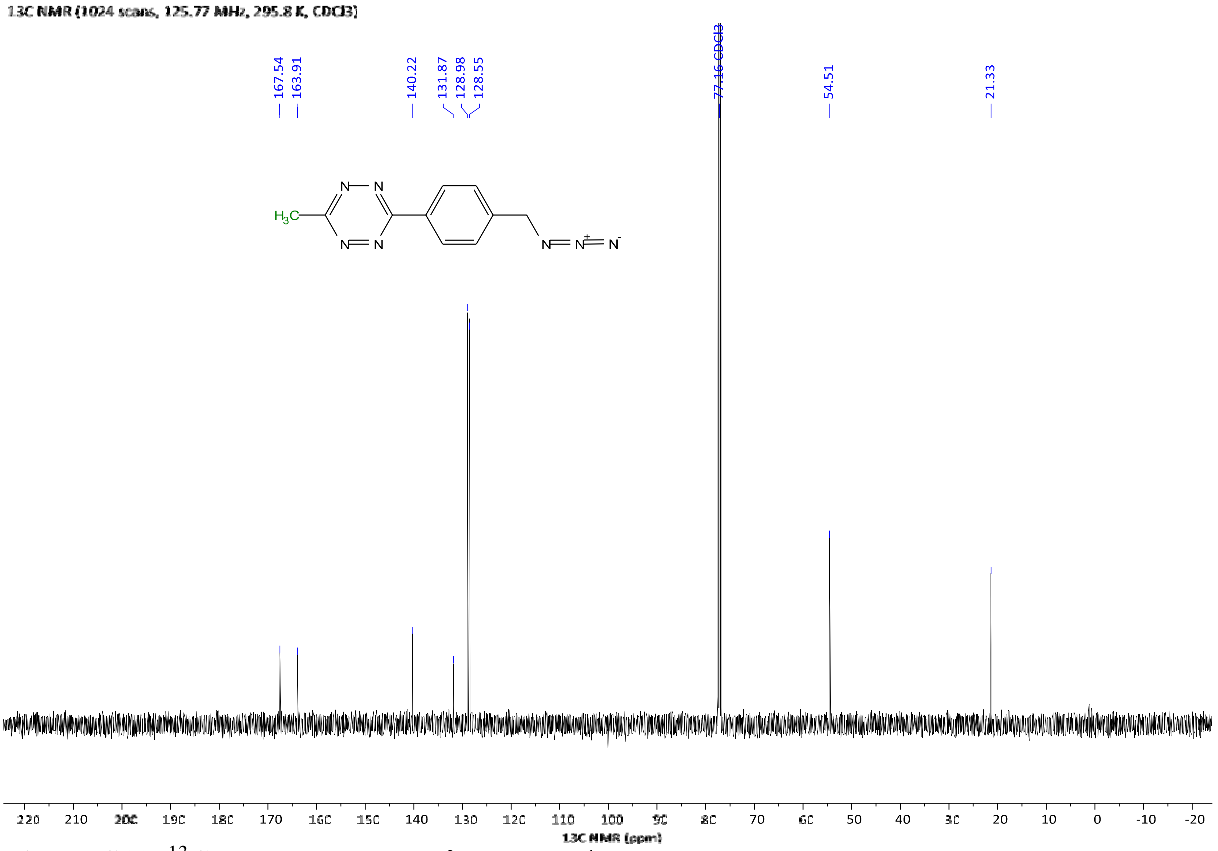

Figure S12. ^{13}C NMR spectrum of compound **7**.

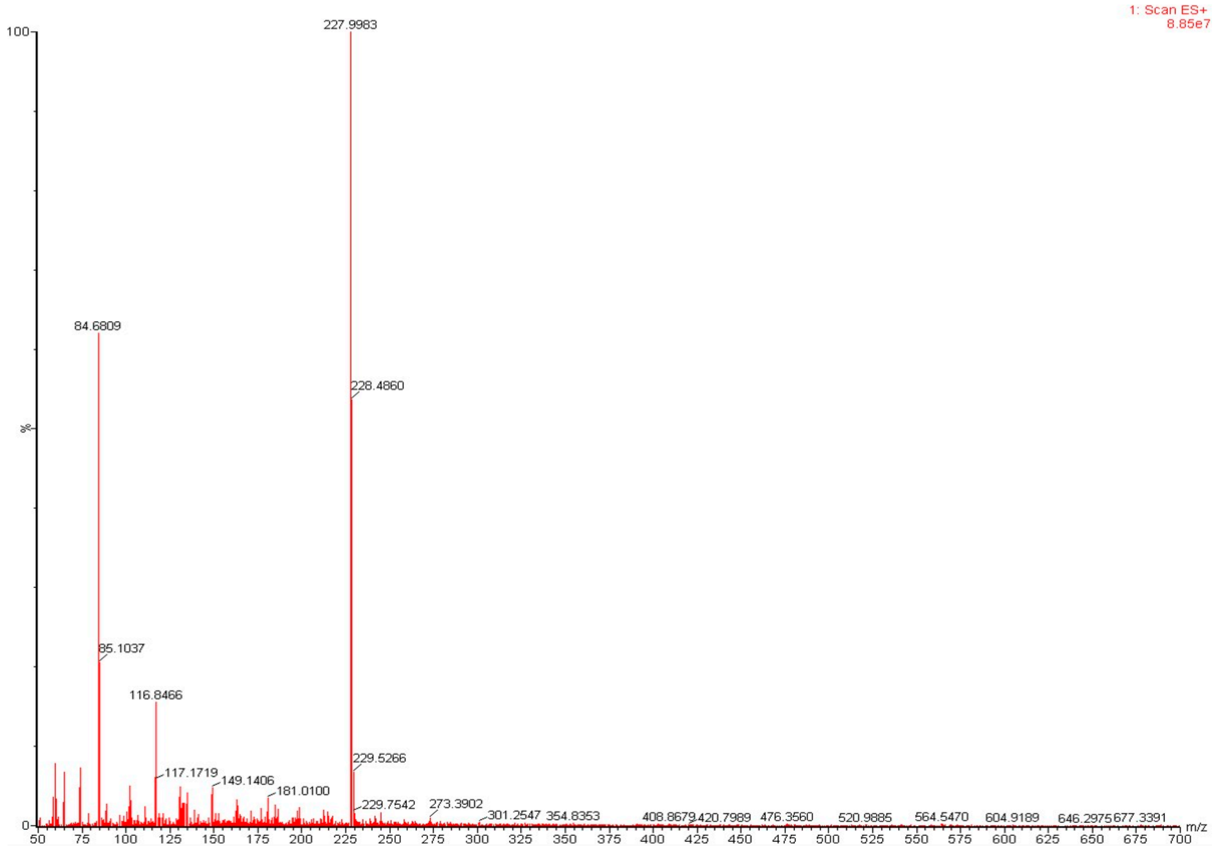

Mass	Calc. Mass	mDa	PPM	DBE	i-FIT	Norm	Conf(%)	Formula
228.0995	228.0998	-0.3	-1.3	9.5	173.5	n/a	n/a	C10 H10 N7

Figure S13. MS and HRMS analysis of compound **7**.

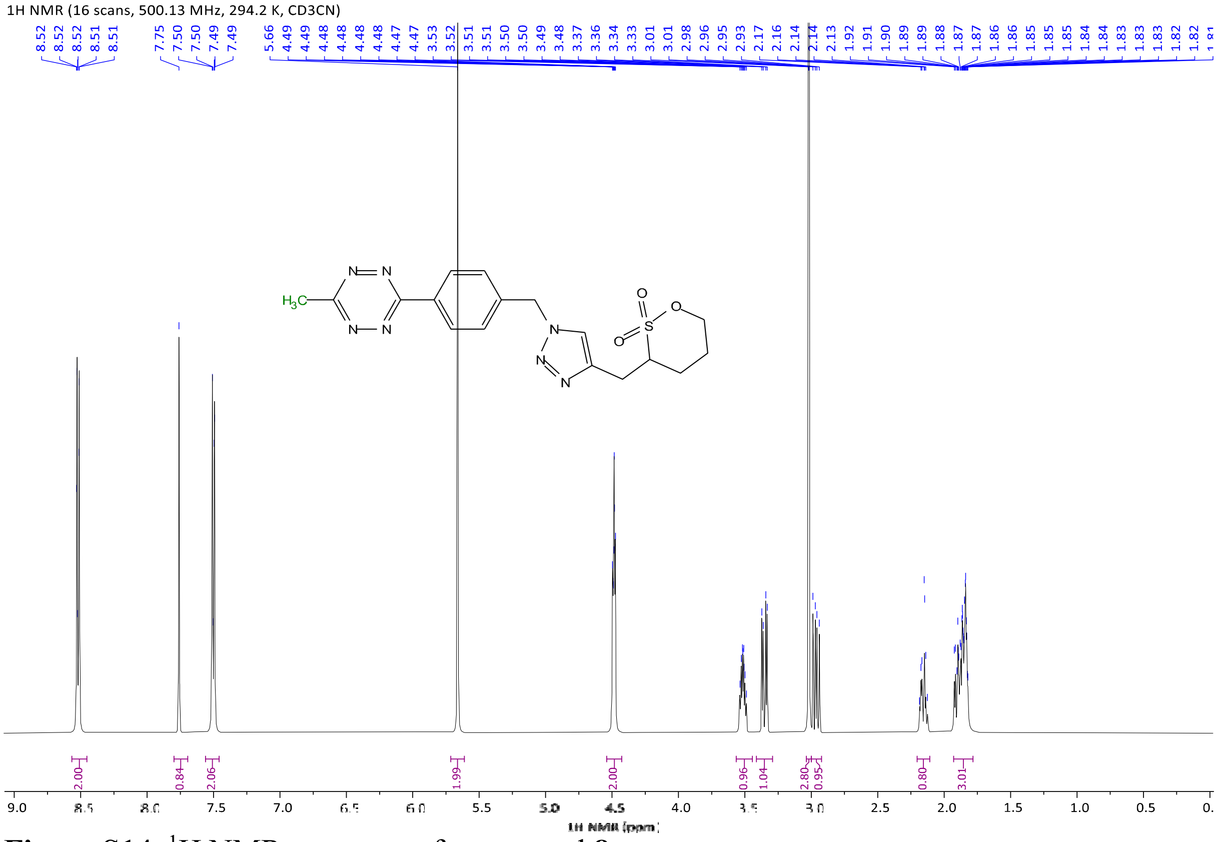

Figure S14. ^{1}H NMR spectrum of compound **8**.

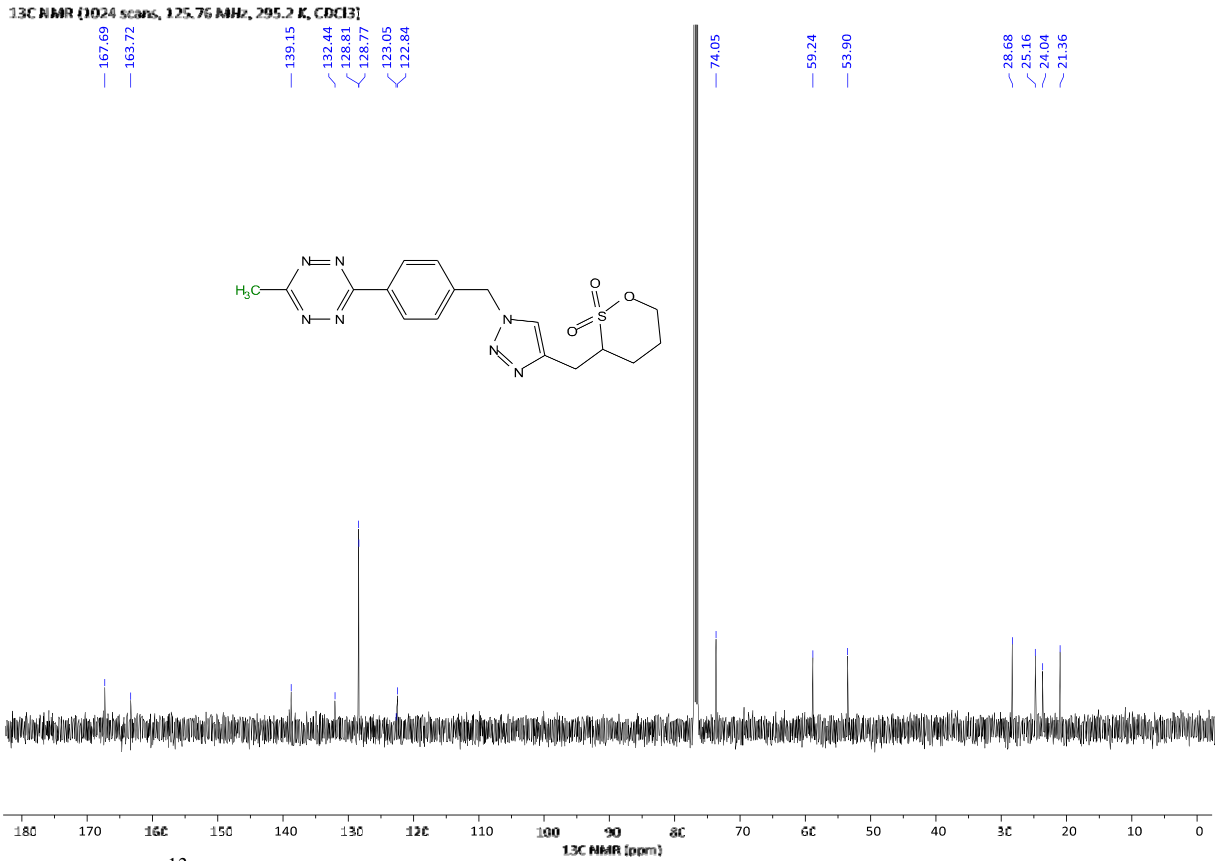

Figure S15. ^{13}C NMR spectrum of compound **8**.

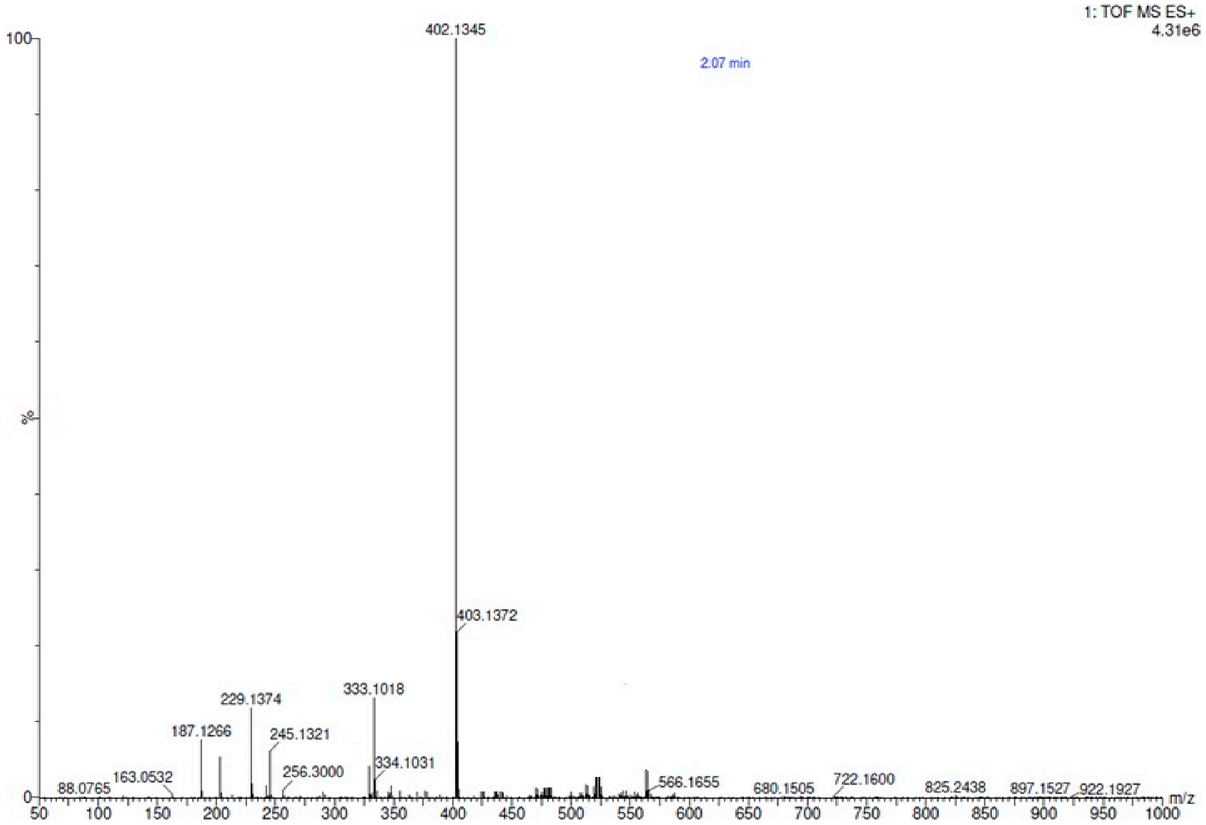

Mass	Calc. Mass	mDa	PPM	DBE	i-FIT	Norm	Conf(%)	Formula
402.1345	402.1348	-0.3	-0.7	11.5	220.8	n/a	n/a	C17 H20 N7 O3 S

Figure S16. MS and HRMS analysis of compound **8**.

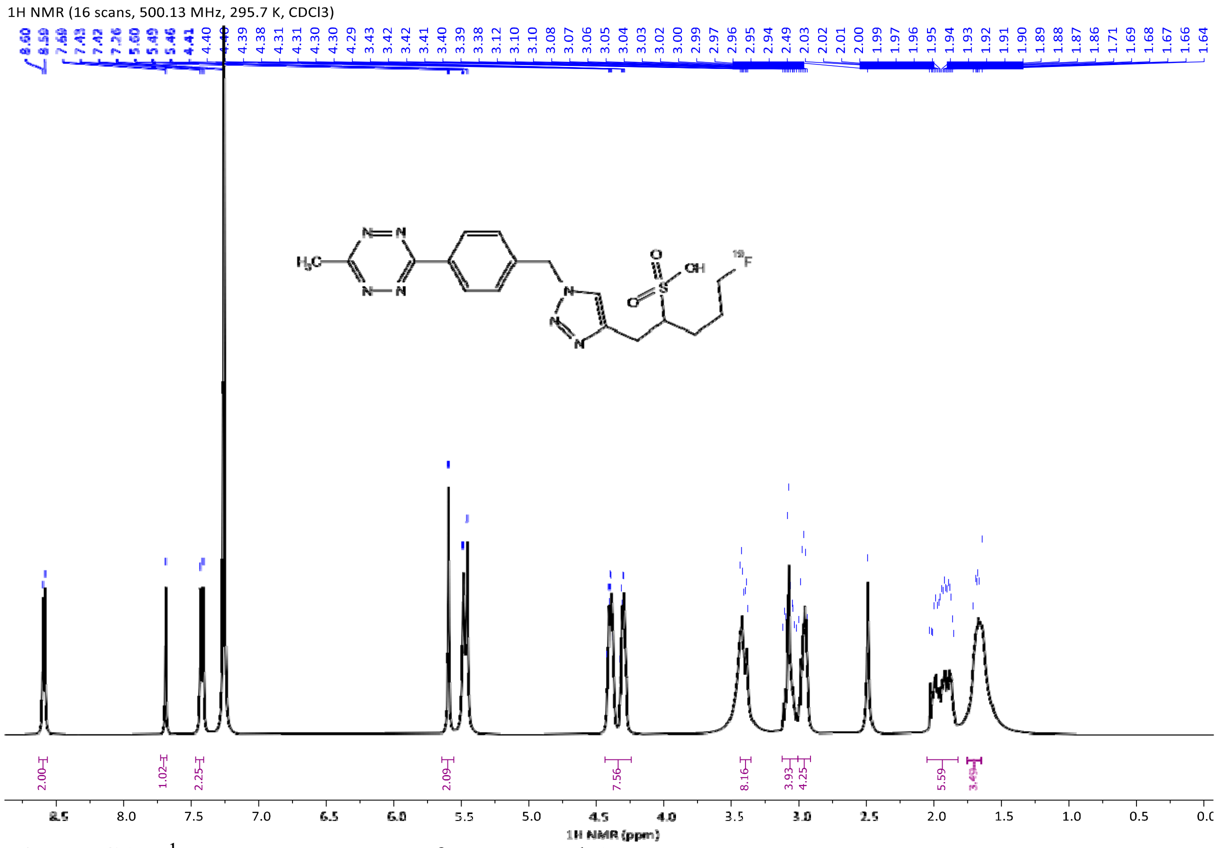

Figure S17. ^{1}H NMR spectrum of compound **1**.

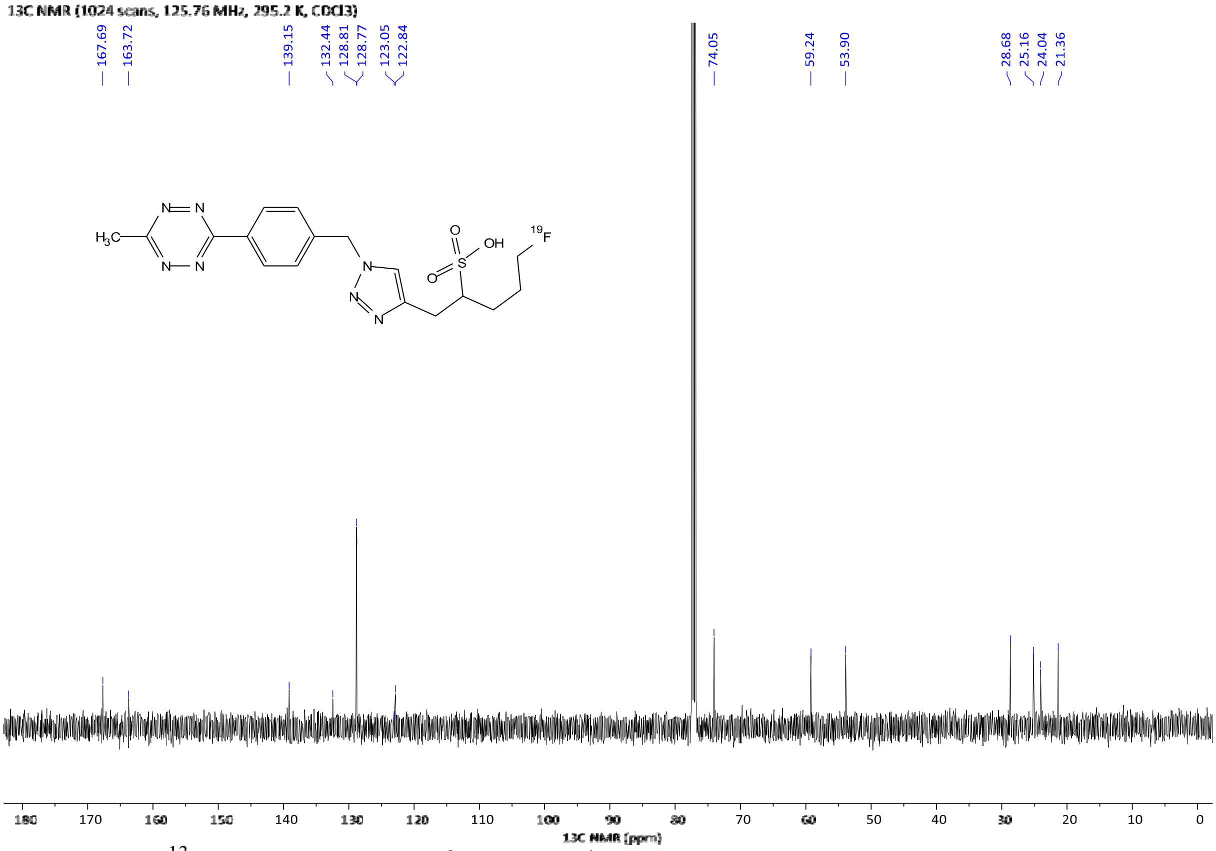

Figure S18. ^{13}C NMR spectrum of compound **1**.

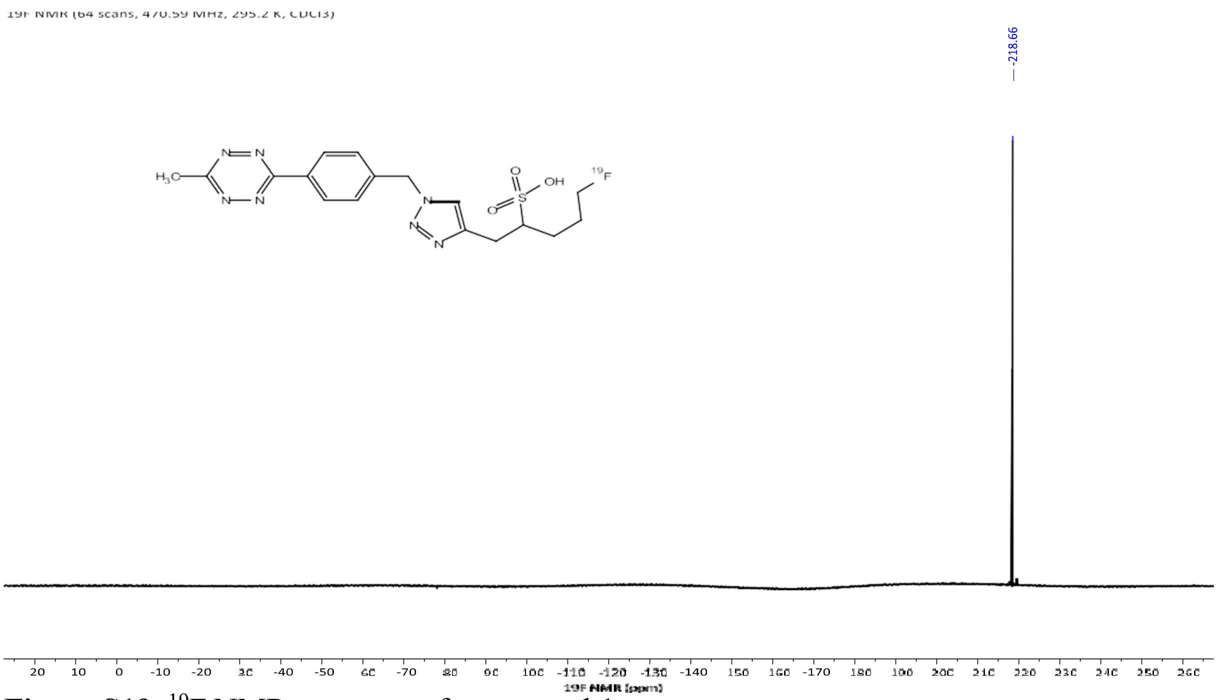

Figure S19. ^{19}F NMR spectrum of compound **1**.

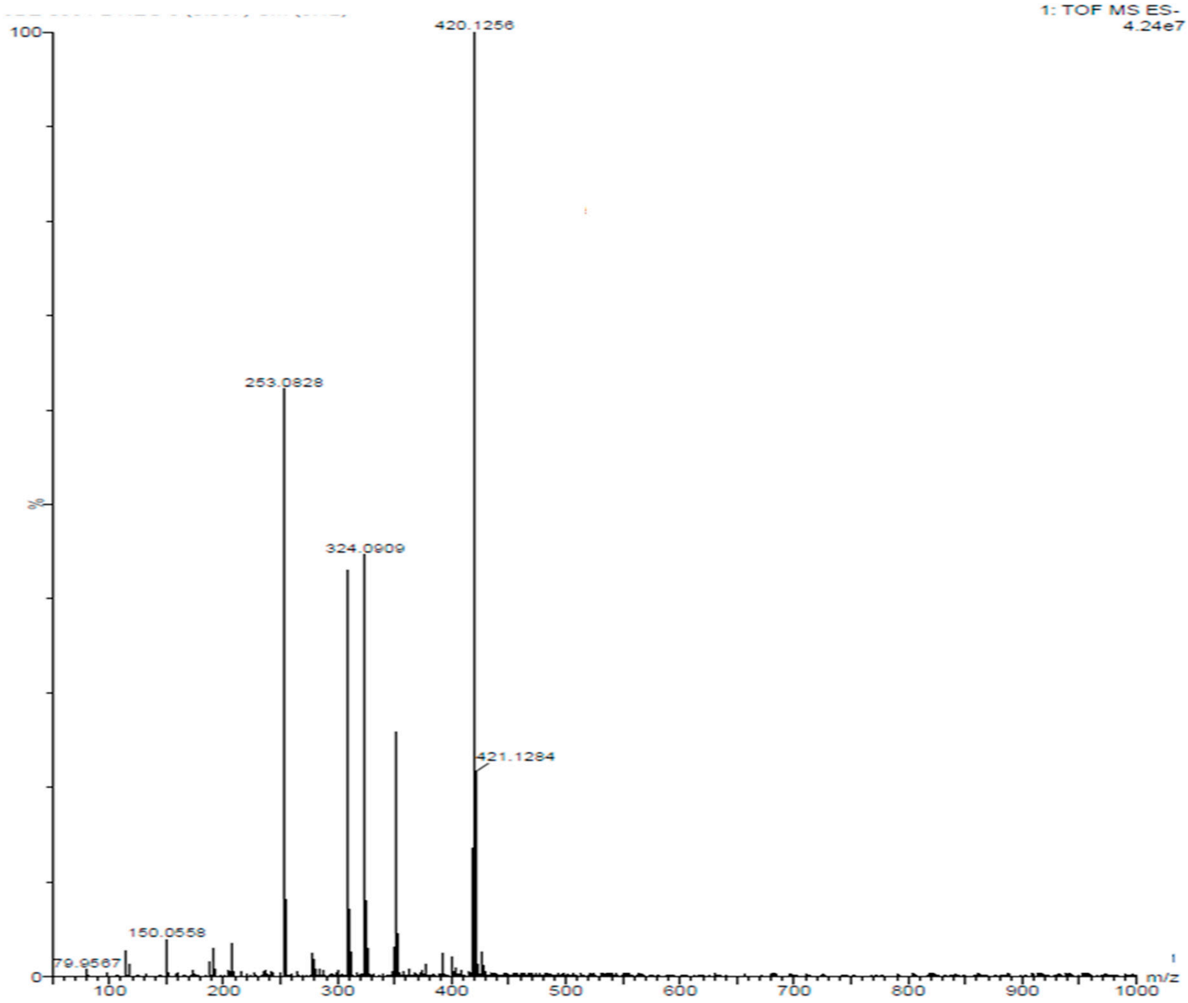

Mass	Calc. Mass	mDa	PPM	DBE	i-FIT	Norm	Conf(%)	Formula
420.1256	420.1254	0.2	0.5	11.5	1631.8	n/a	n/a	C17 H19 N7 O3 S F

Figure S20. MS and HRMS analysis of compound **1**.

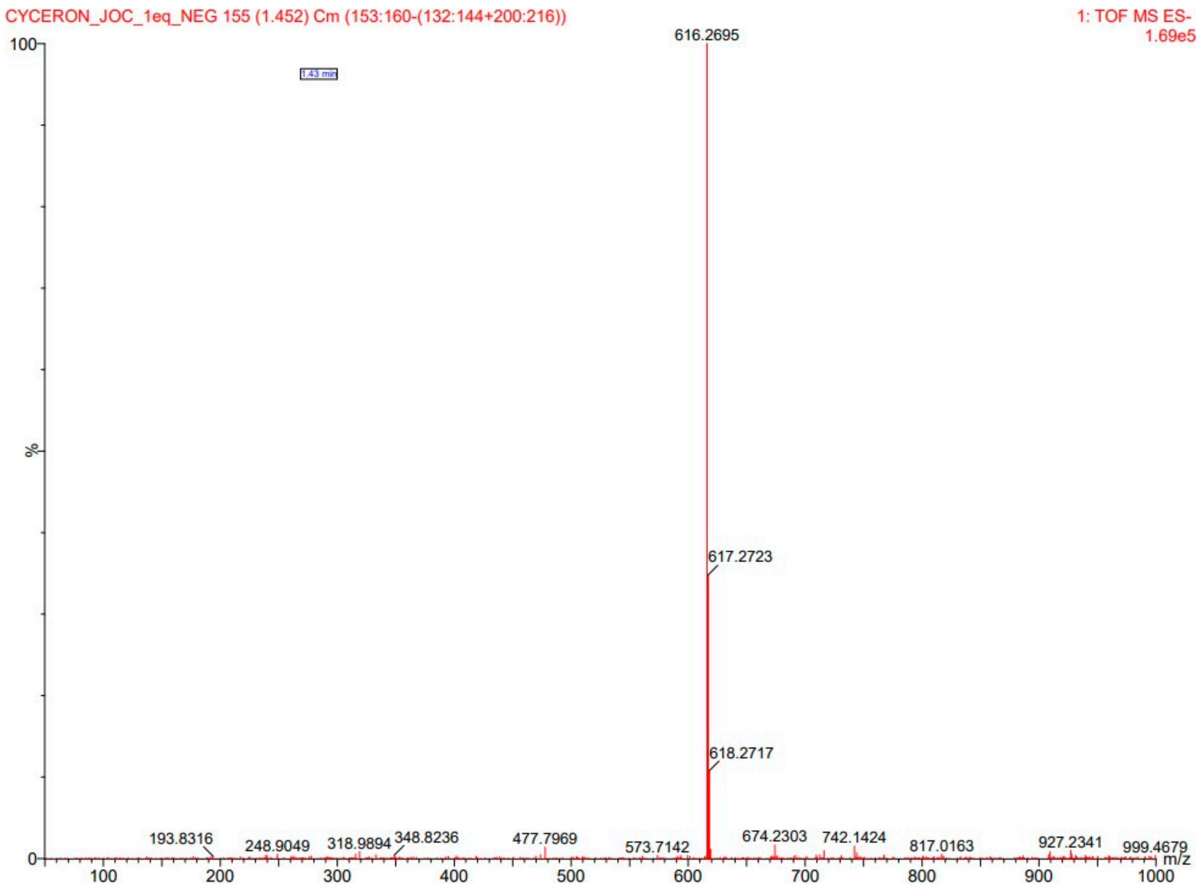

Figure S21. MS analysis of compound **10**.

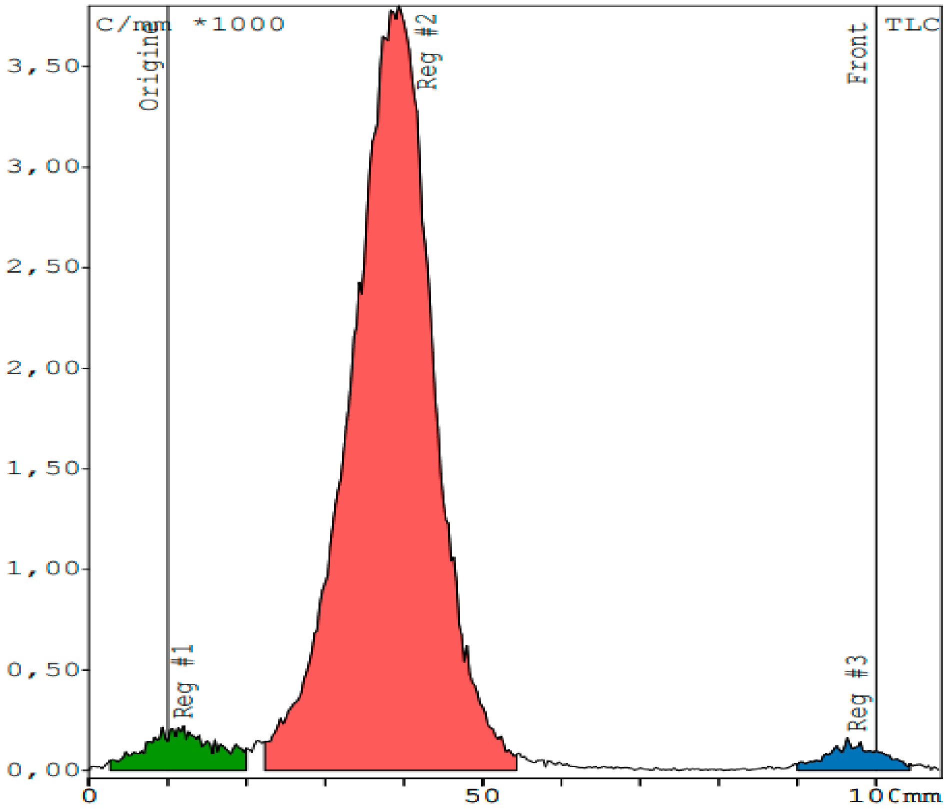

Figure S22. RadioTLC analysis of crude compound [^{18}F]**4** obtained by radiofluorination of **3**.

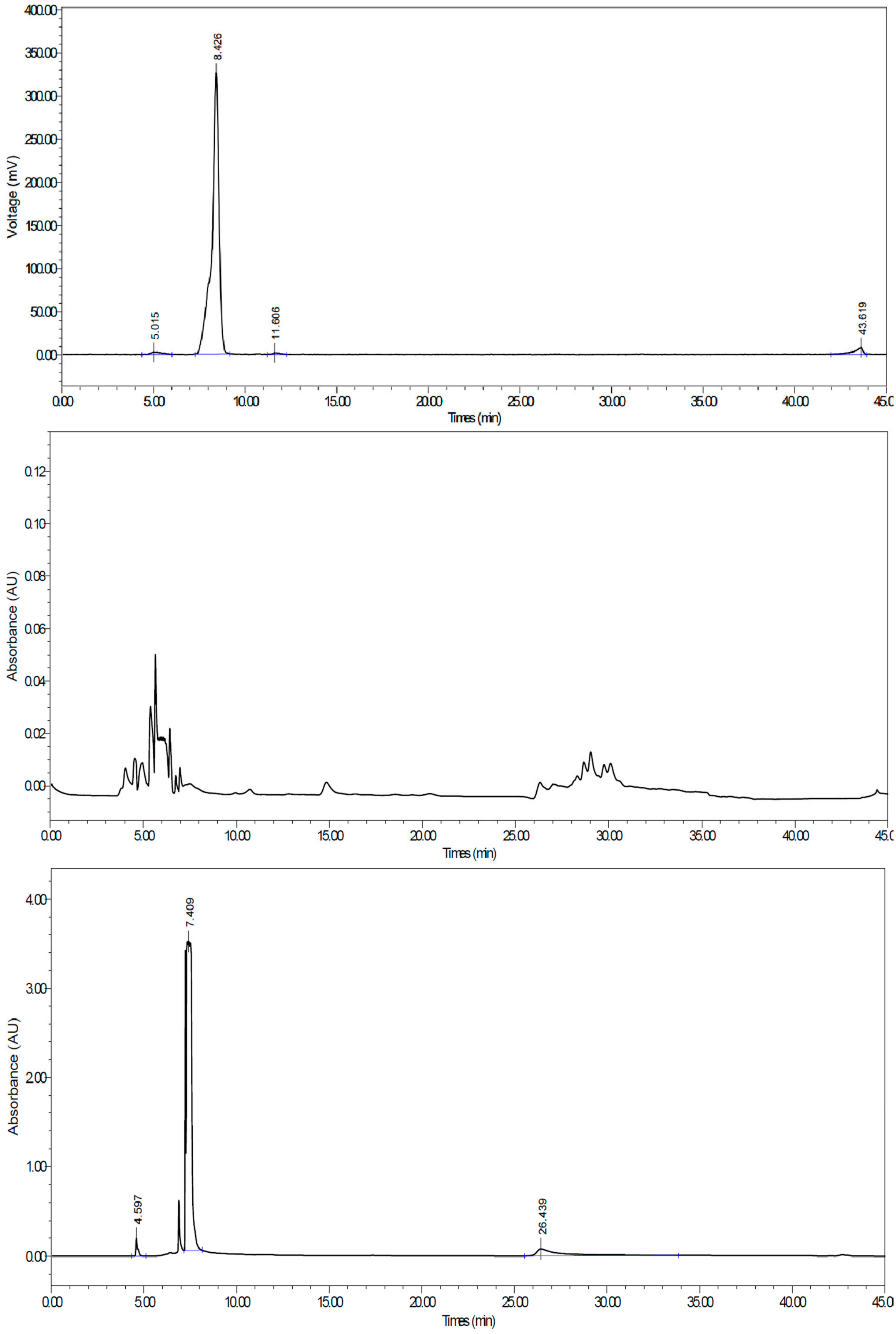

Figure S23. HPLC analysis of crude compound [^{18}F]**4** obtained by radiofluorination of **3**. Top) Gamma detection, Middle) UV detection at 254 nm ; Down) UV Chromatogram of the non radioactive reference compound **4**.
